# Investigating the association of breast cancer and stroke: A two-sample Mendelian randomization study

**DOI:** 10.1097/MD.0000000000035037

**Published:** 2023-09-22

**Authors:** Huiling Qu, Chao He, Haichun Xu, Xiaoyu Sun

**Affiliations:** a Department of Neurology, The General Hospital of Northern Theater Command, Shenyang, China; b Department of Psychiatry, Shenyang Jing'an Mental Health Hospital, Shenyang, Liaoning, P.R. China.

**Keywords:** breast cancer, estrogen receptor, Mendelian randomization, stroke

## Abstract

We conducted a two-sample Mendelian randomization (MR) design to evaluate the causal relation between breast cancer and stroke. Genetic variants associated with breast cancer and stroke were both obtained from genome-wide association study summary data. The single nucleotide polymorphisms were selected as instrumental variables. Effect estimates were primarily evaluated using standard inverse variance weighted. Finally, sensitivity analyses were performed for the detection of potential pleiotropy and heterogeneity in the cause-effect evaluation. There was a causal association of ER-positive breast cancer (odds ratio = 0.11, 95% confidence interval: 0.08–0.16, *P* < .001), and ER-negative breast cancer (odds ratio = 1.04, 95% confidence interval: 1.00–1.07, *P* = .045) with stroke. MR-egger regression revealed that the cause-effect of ER-positive breast cancer (*P* < .001) is drove by the directional horizontal pleiotropy, while there was no directional pleiotropy in the cause-effect of ER-negative breast cancer (*P* = .82). Cochran Q-derived *P*-value from inverse variance weighted (*P* = .27) shown that the cause-effect of ER-negative breast cancer on stroke do not need to consider the effect of heterogeneity. In addition, the leave-one-out analysis showed no influential instruments driving the associations, suggesting robust results for all outcomes. The present MR study reveals that ER negative breast cancer increase the risk of stroke.

## 1. Introduction

In China, stroke is the main cause of death, with the highest mortality rate and disability rate, which brings huge economic burden to the society.^[1]^ Stroke, including hemorrhagic or ischemic stroke, is an acute cerebrovascular disease, which is caused by sudden rupture or blockage of cerebral vessels, leading to insufficient blood supply to brain tissue, eventually leading to brain injury, and seriously reducing the quality of life of patients.^[2]^ Therefore, it is very important to identify each risk factor of stroke and provide guidance for early prevention and treatment.

Breast cancer is a major cause of cancer related mortality in more than 100 countries.^[3]^ Chinese cases account for 12.2% of all newly diagnosed breast cancer cases in the world and 9.6% of breast cancer deaths. Breast cancers that have estrogen receptors are called ER positive cancers. It means that estrogen is fueling the growth of the cancer. However, given high phenotypic and genetic correlation across different subtypes of breast cancer, it remains unclear whether ER positive and ER negative account for the observed associations between breast cancer and stroke. Disentangling the associations of different subtypes of breast cancer and risk of stroke is of great public health and clinical importance.

Mendelian randomization (MR) is an epidemiological approach that can assess a causal effect of exposure on a disease outcome. As a new causality research strategy, MR attracts researchers and is widely used in various research in recent years. In MR analysis, genetic polymorphisms closely related to exposure were used as instrumental variables.^[4]^ Because genetic variants are allocated randomly at conception, the MR design is largely free from confounding and reverse causation. MR can greatly reduce the potential bias of observational studies.

Thus, the aim of this study was to assess the causal relationship between breast cancer and stroke comprehensively using two-sample MR analysis, in which the confounders and biases could be eliminated. To expel the possibility of reverse causality, we performed a reverse MR analysis to examine the influence of liability to stroke on 2 different subtypes of breast cancer.

## 2. Methods

### 2.1. Study design

**Figure 1.**
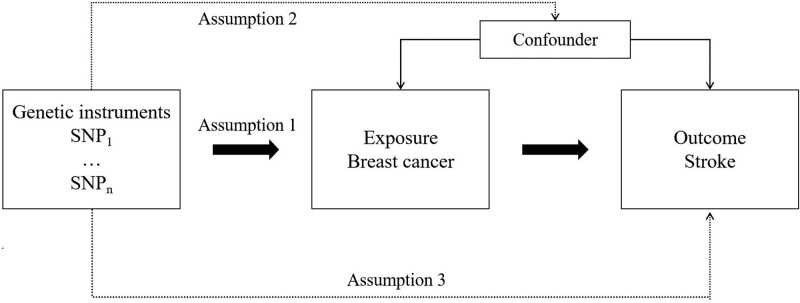
Study design overview. SNP = single-nucleotide polymorphism.

We conducted a two-sample MR design to evaluate the causal relation between breast cancer and stroke (Fig. 1). There is no need for extra ethics approval, because all the analyses were performed based on public genome-wide association study (GWAS) summary-level data.

### 2.2. Selection of instrumental variances for breast cancer

GWAS summary-level data of women for breast cancer and molecular subtypes were downloaded from IEU GWAS database (https://gwas.mrcieu.ac.uk/datasets/). Threshold values were preset (*P*-value < 5*10^−8^; *r*^2^ > 0.001, window size = 10,000 kb) for the selection of single nucleotide polymorphisms (SNPs) significantly associated with the exposure and independent of linkage disequilibrium. In addition, SNPs which are not presented in the outcome GWAS summary-level data or being palindromic with intermediate allele frequency were removed. *F* statistic was calculated for each set of IVs (*F* = *R*^2^(n − *k* − 1)/*k*(1 − *R*^2^). *R*^2^, variance of exposure explained by selected IVs, which is obtained with the “MR Steiger directionality test” function in “Two Sample MR” package; n, sample size of the exposure; *k*, number of instrumental variables.). *F* statistic larger than 10 suggests that the weak instrumental variable bias is unlikely to happen for the IV-exposure association.^[5]^

### 2.3. GWAS summary-level data for stroke

The GWAS summary data for stroke and its clinical subtypes were obtained from the IEU GWAS database (https://gwas.mrcieu.ac.uk/datasets/). Summary statistics for stroke (40,585 cases and 406,111 controls) and ischemic stroke (34,217 cases and 406,111 controls) of European ancestry was obtained.^[6]^ Additionally, the causal role of breast cancer on common etiological subtypes of ischemic stroke such as large artery atherosclerosis (4373 cases and 406,111 controls), cardioembolic stroke (7193 cases and 406,111 controls), and small-vessel stroke (5386 cases and 192,662 controls) was also evaluated.^[7]^ Ischemic stroke subtypes were classified based on the Trial of Org 10172 in Acute Stroke Treatment criteria.^[8]^ Table S1, Supplemental Digital Content, http://links.lww.com/MD/J883 shows the detailed descriptions of the data sources.

### 2.4. Statistical analyses

Exposure data and outcome data were combined through the “harmonise_data” function in “TwoSampleMR” R package. Several MR approaches were adopted for the evaluation of cause-effect from breast cancer, ER-positive breast cancer, and ER-negative breast cancer on stroke, ischemic stroke, ischemic stroke (cardioembolic), ischemic stroke (small-vessel), and ischemic stroke (large artery atherosclerosis), respectively, which includes inverse variance weighting (IVW), MR-Egger as well as weighted median. These approaches were built based on different hypothesis of consistency and exhibited different strengths and weaknesses on evaluating cause-effect. The IVW approach can evaluate the cause-effect based on the premise that there is no horizontal pleiotropy in IVs and exhibit the most robust efficiency.^[9,10]^ In this study, we used default random effect IVW approach. The MR-Egger approach can evaluate the cause-effect based on premise that the IVs should fulfill the Instrument Strength Independent of Direct Effect assumption.^[11]^ The weighted median approach was also used to complement the evaluation. We chose the result from IVW to support our main conclusion as it can provide the most robust evaluation of cause-effect. If inconsistency occur in the cause-effect evaluation, the *P*-value for filtering exposure associated SNPs will be tighten.

Sensitivity analyses were performed for the detection of potential pleiotropy and heterogeneity in the cause-effect evaluation. Cochran Q-derived *P*-value from IVW and MR-egger approach is used for the detection of heterogeneity. MR-egger regression can evaluate the potential pleiotropic effects for the instrumental SNPs. If significant intercept is identified by MR-egger regression, the cause-effect is drove by the directional horizontal pleiotropy.^[12]^ MR-PRESSO method also can estimate the horizontal pleiotropy and correct it through removing outliner SNPs.^[13]^ The number of distributions in MR-PRESSO analysis was set to 3000. In addition, leave-one-out analysis was performed to detect single SNP which can drive the result.

All the analyses in this study were based on software R 4.2.0. “TwoSampleMR” package 0.5.6 version which was developed by Neil Martin Davies was used for MR analysis and sensitivity analysis.^[10]^

## 3. Results

After LD clumping, we identified 140, 106, and 37 SNPs that were robustly and independently associated with breast cancer, ER-positive breast cancer, and ER-negative breast cancer, respectively (*P* < 5 × 10^–8^). These 283 SNPs had *F*-statistics ranging from 29.73 to 1447.04, suggesting that there was limited evidence of weak instrument bias. Information on the instrumental variables is presented in Table S2, Supplemental Digital Content, http://links.lww.com/MD/J884.

The results of the main MR analyses are shown in Table 1. There was causal association of ER-positive breast cancer [odds ratio (OR) = 0.11, 95% confidence interval [CI]: 0.08–0.16, *P* < .001], and ER-negative breast cancer (OR = 1.04, 95% CI: 1.00–1.07, *P* = .045) with stroke. Also, there was no causal association of breast cancer with stroke (OR = 0.81, 95% CI: 0.57–1.14, *P* = .224), ischemic stroke (OR = 1.01, 95% CI: 0.97–1.04, *P* = .708), cardioembolic stroke (OR = 1.04, 95% CI: 0.98–1.10, *P* = .185), small vessel stroke (OR = 1.01, 95% CI: 0.95–1.08, *P* = .729), large artery stroke (OR = 1.05, 95% CI: 0.99–1.12, *P* = .133) and there was no causal association of ER-positive breast cancer with ischemic stroke (OR = 1.00, 95% CI: 0.95–1.05, *P* = .892), cardioembolic stroke (OR = 1.03, 95% CI: 0.97–1.10, *P* = .315), small vessel stroke (OR = 1.03, 95% CI: 0.96–1.09, *P* = .424), large artery stroke (OR = 0.89, 95% CI: 0.77–1.04, *P* = .142), and there was no causal association of ER-negative breast cancer with ischemic stroke (OR = 1.04, 95% CI: 0.99–1.08, *P* = .101), cardioembolic stroke (OR = 1.04, 95% CI: 0.96–1.12, *P* = .328), small vessel stroke (OR = 0.97, 95% CI: 0.90–1.04, *P* = .402), large artery stroke (OR = 1.03, 95% CI: 0.92–1.14, *P* = .641). As shown in Table 2, reverse MR analysis showed that there was causal association of ischemic stroke with ER-positive breast cancer (OR = 0.89, 95% CI: 0.79–1.00, *P* = .06) and no causal associations were found in other stroke and subtypes with breast cancer, ER-positive breast cancer and ER-negative breast cancer.

**Table 1:** Mendelian randomization for the association of breast cancer with stroke.

Exposure	Outcome	Association method				Heterogeneity		Pleiotropy	
			OR	95 % CI	*P*	*Q* statistic	*P*	Intercept	*P*
Breast cancer	Stroke	Inverse variance weighted	0.808	0.573–1.139	.224	30,568.31	0	–	–
		MR Egger	1.879	0.949–3.720	.073	28,868.97	0	−0.07	.006
		Weighted median	1.028	0.989–1.067	.158	–	–	–	–
ER-positive breast cancer	Stroke	Inverse variance weighted	0.112	0.080–0.156	<.001	60,308.27	0	–	–
		MR Egger	3.239	1.503–6.977	.003	32,028.47	0	−0.236	<.001
		Weighted median	1.022	0.986–1.059	.241	–	–	–	–
ER-negative breast cancer	Stroke	Inverse variance weighted	1.036	1.001–1.073	.045	36.32	.274	–	–
		MR Egger	1.048	0.949–1.156	.362	36.26	.237	−0.07	.817
		Weighted median	1.032	0.982–1.084	.21	–	–	–	–
Breast cancer	Ischemic stroke	Inverse variance weighted	1.006	0.974–1.040	.708	174.84	.006	–	–
		MR Egger	1.008	0.942–1.078	.822	174.84	.005	0	.961
		Weighted median	1.008	0.982–1.061	.766	–	–	–	–
ER-positive breast cancer	Ischemic stroke	Inverse variance weighted	0.997	0.950–1.045	.892	300.96	<.001	–	–
		MR Egger	0.999	0.909–1.097	.979	300.96	<.001	0	.961
		Weighted median	1.005	0.956–1.057	.843	–	–	–	–
ER-negative breast cancer	Ischemic stroke	Inverse variance weighted	1.035	0.993–1.079	.101	28.79	.63	–	–
		MR Egger	1.063	0.943–1.198	.328	28.58	.591	−0.003	.65
		Weighted median	1.041	0.981–1.104	.184	–	–	–	–
Breast cancer	Ischemic stroke (cardioembolic)	Inverse variance weighted	1.037	0.983–1.095	.185	157.09	.06	–	–
		MR Egger	1.079	0.966–1.206	.18	156.31	.058	−0.003	.422
		Weighted median	1.031	0.945–1.126	.488	–	–	–	–
ER-positive breast cancer	Ischemic stroke (cardioembolic)	Inverse variance weighted	1.032	0.971–1.097	.315	156.32	<.001	–	–
		MR Egger	1.088	0.964–1.228	.174	154.68	<.001	-0.005	.32
		Weighted median	1.026	0.945–1.114	.541	–	–	–	–
ER-negative breast cancer	Ischemic stroke (cardioembolic)	Inverse variance weighted	1.041	0.961–1.127	.328	39.83	.161	–	–
		MR Egger	1.256	1.007–1.566	.052	36.13	.241	−0.02	.084
		Weighted median	1.102	0.988–1.228	.081	–	–	–	–
Breast cancer	Ischemic stroke (small-vessel)	Inverse variance weighted	1.011	0.949–1.078	.729	182.63	<.001	–	–
		MR Egger	0.999	0.875–1.140	.986	182.55	<.001	0.001	.832
		Weighted median	1.033	0.946–1.128	.469	–	–	–	–
ER-positive breast cancer	Ischemic stroke (small-vessel)	Inverse variance weighted	1.026	0.963–1.093	.424	143.02	<.001	–	–
		MR Egger	1.078	0.950–1.223	.246	141.7	<.001	−0.005	.378
		Weighted median	1.04	0.968–1.119	.284	–	–	–	–
ER-negative breast cancer	Ischemic stroke (small-vessel)	Inverse variance weighted	0.968	0.898–1.044	.402	30.84	.324	–	–
		MR Egger	1.039	0.855–1.263	.702	30.17	.306	−0.008	.448
		Weighted median	0.958	0.863–1.064	.427	–	–	–	–
Breast cancer	Ischemic stroke (large artery atherosclerosis)	Inverse variance weighted	1.05	0.985–1.119	.133	156.03	.067	–	–
		MR Egger	1.046	0.918–1.192	.504	156.03	.06	0	.944
		Weighted median	1.02	0.926–1.124	.687	–	–	–	–
ER-positive breast cancer	Ischemic stroke (large artery atherosclerosis)	Inverse variance weighted	0.894	0.770–1.038	.142	683.12	<.001	–	–
		MR Egger	1.237	0.924–1.656	.157	639.76	<.001	−0.032	.014
		Weighted median	0.988	0.904–1.080	.787	–	–	–	–
ER-negative breast cancer	Ischemic stroke (large artery atherosclerosis)	Inverse variance weighted	1.026	0.920–1.144	.641	53.91	.009	–	–
		MR Egger	0.894	0.663–1.206	.469	52.32	.01	0.015	.339
		Weighted median	1.091	0.962–1.237	.174	–	–	–	–

CI = confidence interval; IVW = inverse variance weighted; OR = odds ratio; SE = standard error.

**Table 2:** Mendelian randomization for the association of stroke with breast cancer.

Exposure	Outcome	Association method			Heterogeneity			Pleiotropy	
			OR	95 % CI	*P*	*Q* statistic	*P*	Intercept	*P*
Stroke	Breast cancer	Inverse variance weighted	0.89	0.789–1.003	.055	50.77	<.001	–	–
		MR Egger	1.115	0.495–2.515	.796	49.61	<.001	−0.014	.591
		Weighted median	0.956	0.863–1.058	.383	–	–	–	–
	ER-positive breast cancer	Inverse variance weighted	0.89	0.787–1.006	.063	37.79	.001	–	–
		MR Egger	1.268	0.556–2.892	.582	35.79	.001	−0.021	.409
		Weighted median	0.945	0.841–1.063	.347	–	–	–	–
	ER-negative breast cancer	Inverse variance weighted	0.891	0.768–1.035	.132	24.04	.045	–	–
		MR Egger	1.479	0.548–3.991	.454	22.29	.051	−0.03	.33
		Weighted median	0.841	0.709–0.997	.046	–	–	–	–
Ischemic stroke	Breast cancer	Inverse variance weighted	0.889	0.788–1.002	.055	20.44	.005	–	–
		MR Egger	1.448	0.667–3.143	.385	16.23	.013	−0.037	.258
		Weighted median	0.92	0.823–1.028	.143	–	–	–	–
	ER-positive breast cancer	Inverse variance weighted	0.886	0.792–0.992	.036	12.63	.082	–	–
		MR Egger	1.462	0.720–2.970	.334	9.52	.146	−0.038	.211
		Weighted median	0.913	0.808–1.031	.144	–	–	–	–
	ER-negative breast cancer	Inverse variance weighted	0.867	0.735–1.023	.091	11.65	.113	–	–
		MR Egger	1.111	0.342–3.610	.866	11.32	.079	−0.019	.691
		Weighted median	0.828	0.695–0.985	.033	–	–	–	–
Ischemic stroke (cardioembolic)	Breast cancer	Inverse variance weighted	0.979	0.939–1.021	.317	0.41	.939	–	–
		MR Egger	0.996	0.899–1.102	.94	0.28	.869	−0.003	.757
		Weighted median	0.982	0.938–1.028	.443	–	–	–	–
	ER-positive breast cancer	Inverse variance weighted	0.963	0.913–1.015	.16	3.4	.333	–	–
		MR Egger	0.952	0.813–1.115	.603	3.36	.186	0.002	.892
		Weighted median	0.969	0.913–1.029	.305	–	–	–	–
	ER-negative breast cancer	Inverse variance weighted	0.956	0.886–1.031	.244	0.91	.823	–	–
		MR Egger	0.94	0.781–1.130	.577	0.87	.648	0.003	.86
		Weighted median	0.957	0.879–1.041	.305	–	–	–	–
Ischemic stroke (small-vessel)	Breast cancer	Inverse variance weighted	–	–	–	–	–	–	–
		MR Egger							
		Weighted median							
	ER-positive breast cancer	Inverse variance weighted	–	–	–	–	–	–	–
		MR Egger							
		Weighted median							
	ER-negative breast cancer	Inverse variance weighted	–	–	–	–	–	–	–
		MR Egger							
		Weighted median							
Ischemic stroke (large artery atherosclerosis)	Breast cancer	Inverse variance weighted	1.028	0.980–1.079	.251	1.67	.644	–	–
		MR Egger	0.946	0.704–1.271	.746	1.35	.509	0.016	.629
		Weighted median	1.032	0.975–1.092	.282	–	–	–	–
	ER-positive breast cancer	Inverse variance weighted	1.02	0.964–1.080	.49	1.08	.781	–	–
		MR Egger	0.944	0.665–1.341	.78	0.89	.64	0.014	.705
		Weighted median	1.015	0.949–1.086	.667	–	–	–	–
	ER-negative breast cancer	Inverse variance weighted	1.066	0.953–1.192	.262	4.93	.177	–	–
		MR Egger	0.883	0.393–1.981	.791	4.45	.108	0.035	.689
		Weighted median	1.059	0.952–1.177	.292	–	–	–	–

CI = confidence interval; IVW = inverse variance weighted; OR = odds ratio; SE,= standard error.

MR-egger regression revealed that the cause-effect of ER-positive breast cancer (*P* < .001) is drove by the directional horizontal pleiotropy, while there was no directional pleiotropy in the cause-effect of ER-negative breast cancer (*P* = .82) (Table 2). Cochran Q-derived *P*-value from IVW (*P* = .27) shown that the cause-effect of ER-negative breast cancer on stroke do not need to consider the effect of heterogeneity. In addition, the leave-one-out analysis showed no influential instruments driving the associations, suggesting robust results for all outcomes (Fig. S1, Supplemental Digital Content, http://links.lww.com/MD/J885) and scatter plots of the IVW and MR-egger approach in 27 causal associations with the breast cancer and stroke can be seen in Figure S2, Supplemental Digital Content, http://links.lww.com/MD/J886.

## 4. Discussion

The present study confirmed the causal effects of breast cancers with different ERs on stroke. Results of two-sample MR analyses showed that ER positive breast cancer has significant negative correlation with stroke risk and ER negative breast cancer increase the risk of stroke. This study did not observe any associations of breast cancer and different subtypes of with ischemic stroke and subtypes.

Some cases series and retrospective study has showed that breast cancer is a risk factor for stroke.^[14]^ Nilsson et al performed an observational study, and, from comparing breast cancer patients with stroke to patients without, drew the conclusion that the risk of stroke was increased in patients with breast cancer [RR = 1.12 95% CI = (1.07, 1.17)]. It seemed that breast cancer was a risk factor for stroke, especially ischemic stroke.^[15]^ A system review published by Zhang et al revealed that breast cancer seems a risk factors for stroke.^[16]^ However, most of above studies were cases series, retrospective, and observational studies, thus the causal relationship between breast cancer and stroke or ischemic stroke was not yet clear. In the present work, we used two-sample MR to explore the causal relationship between breast cancer and stroke, in which the confounders and biases could be eliminated, and reverse causality could be avoided. On the one hand, our findings of the MR investigation are overall in line with previous studies on breast cancer in relation to stroke. Our results showed that ER negative breast cancer is associated with an increased risk of stroke. On the other hand, we revealed that ER positive breast cancer has significant negative correlation with stroke risk, which is not consistent with the present observational studies.^[17]^

Most research on cancer and stroke shows that cancer is a risk factor for stroke because of hypercoagulability, venous-to-arterial embolism, nonbacterial thrombotic endocarditis, direct compression of blood vessels by tumor, radiotherapy and chemotherapy.^[18]^ Nevertheless, the specific mechanisms of stroke in patients with breast cancer are not fully clear. In hemorrhagic stroke, breast cancer is one of the most common solid tumors because of their high incidence rate, brain metastasis, neovascularization, necrosis and vascular invasion in the population.^[19]^ But in ischemic stroke, one possible explanation is that cancer can cause hypercoagulability through changes in circulating particles, secretion of proliferation factors, platelet activity and endothelial function.^[20,21]^ Additionally, several cancer treatments, particularly platinum-based compounds, may increase thrombotic risk.^[21,22]^Our results showed that ER negative breast cancer is associated with an increased risk of stroke. The reason may be related to the mechanisms reported above.

Presently, the mechanisms of the relationship between breast cancer and stroke have not been fully established, as there were limited studies with regard to the mechanisms. The roles of estrogen include growth and maintenance of the skeleton, and normal functioning of both the cardiovascular and central nervous systems. In addition to its role in normal physiology, estrogen is also associated with many diseases, especially breast cancer.^[23]^ In women, epidemiological studies have shown that long-term use of oral contraceptives containing estrogen or hormone replacement therapy will increase the incidence rate of breast cancer.^[24,25]^ In women, early menarche or late menopause will increase the risk of breast cancer.^[26,27]^ The molecular mechanism by which estrogen increases the risk of breast cancer remains unclear. At the physiological level, estrogen is not the cause of breast cancer.^[28]^ However, either surgical oophorectomy or LH–RH analogs (such as goserelin, leuprorelin, and buserelin) can be used to treat breast cancer by reducing the level of circulating estrogen.^[29,30]^ When breast cancer patients were grouped according to estrogen level, the recurrence free survival rate was significantly reduced in the groups with low estrogen level (10–49 fmol/mg protein) compared with high estrogen level (>50 fmol/mg protein). The overall survival benefit of women with lower estrogen level was slightly higher than that of women with higher estrogen level (*P* = .046).^[31]^ This indicates that estrogen level can affect the occurrence and prognosis of breast cancer. Postmenopausal women with high estradiol levels have an increased risk of breast cancer^[32]^ and postmenopausal estrogenic activity is a strong determinant of the incidence of ER positive breast cancer.^[33]^ Not all breast cancer is related to estrogen, but ER positive cancer is the most common type of breast cancer. ER positive breast cancer means that estrogen promotes the growth of cancer cells. Notably, estrogen has been widely proven to be effective in protecting the brain from experimental stroke.^[34–38]^ Many properties of estrogen may be beneficial to human vascular stroke, and these properties have been positively characterized in animal and related cell models.^[39]^ Compared with men, women have a lower incidence rate of stroke in most of their lives, which is due to the protective effect of gonadal hormones, especially estrogen. Due to the low incidence rate of stroke observed in premenopausal women, and the preclinical evidence that estrogen has neuroprotective and anti-inflammatory effects. However, as women age, they are disproportionately affected by stroke, which is consistent with the loss of postmenopausal estrogen.^[40]^ The risk of stroke in elderly women is higher than that in men. It is clear that in some cases, estrogen may have proinflammatory effects.^[40]^ However, high doses of estrogen are associated with elevated levels of serum C-reactive protein, which is a marker of vascular risk.^[41–43]^ During pregnancy, estrogen levels rise steadily and cause hypercoagulable state, which may be the reason for the increased risk of stroke in perinatal women.^[44,45]^ More research is needed to understand the potential mechanism of estrogen fluctuations or changes and stroke in the future. In all, estrogen is a “double-edged sword,” which not only has neuroprotective effects in most cases, but also can cause diseases such as breast cancer, which may explain ER positive breast cancer has significant negative correlation with stroke risk to some extent in our study. In addition, pleiotropy might remain, indicating that there are other factors involved in the ER positive breast cancer and stroke.

Although there is a large amount of evidence in the literature to link the occurrence of stroke with malignant tumors, the reverse MR analysis showed that there were not any associations of stroke on breast cancer and subtypes. Our analysis only show that ischemic stroke is negatively correlated with ER positive breast cancer, but the retrograde relationship and underlying mechanism between cancer and stroke remains to be confirmed. Whether stroke will cause cancer, or whether it may be an early manifestation of cancer, remains to be determined.^[46,47]^

Since our results showed that ER negative breast cancer might increase the risk of stroke, much more attention should be paid to patients with ER negative breast cancer, with regard to the prevention of stroke. Given the epidemiological evidence that estrogenic stimulation can increase risk of ER positive breast cancer, we should begin to pay attention to the close relationship between estrogen and breast cancer. But there is no denying the protective effect of estrogen on the nervous system in most cases. Firstly, a better understanding of the comparative role of breast cancer in stroke not only facilitates a clearer perception of the underlying pathophysiology of stroke, but also helps to find statistically significant predictors for cancer and a possible link between breast cancer and stroke. Secondly, the findings can provide an evidence basis in guiding the prevention and treatment of stroke among breast cancer population.^[15]^ Thirdly, such investigation will help unify the guidelines concerning identify cancer survivors at elevated risk of stroke.^[48]^ But our study did not observe any associations of breast cancer and different subtypes with ischemic stroke and subtypes. More research may be needed to explain in the future.

There were strengths to this study. Almost all previous studies on the causal effect of breast cancer on stroke were observational or retrospective studies, with only a few cases, in which bias and confounding factors cannot be eliminated. MR analysis was used to evaluate this potential causality comprehensively, in which reduces confusion and reverse causality. We used updated genetic instruments for different subtypes of breast cancer, thereby ensuring an adequate power in analysis. To the best of our knowledge, the study is the first one investigating the association of ischemic stroke and breast cancer using MR analysis. It is worth noting that our MR study also has limitations. Firstly, we were unable to perform stages of breast cancer since the individual-level data were not available. Secondly, we could not adjust for the use of treatments and some breast cancer treatments have been associated with increased stroke risk, due to no access to individual-level data. Thirdly, we limited our research population to individuals of European descent, and it is unclear whether this causal relationship applies to other lineages. Moreover, even when the similarity assumption of sex distribution between gene-exposure and gene-outcome associations is violated, MR approach could still provide evidence on whether a causal association exists despite not necessarily on the precise magnitude of the causal effect.^[49]^ Future work on such topics may be focused on different sex categories.

## 5. Conclusions

In summary, this two-sample MR analysis showed that ER negative breast cancer patients may have a higher risk of stroke, so more attention should be paid to stroke prevention in these patients.

## Author contributions

**Data curation:** Haichun Xu.

**Supervision:** Xiaoyu Sun.

**Writing – original draft:** Huiling Qu.

**Writing – review & editing:** Chao He.

## Supplementary Material








