# Supplementary material for: Investigating the association of breast cancer and stroke: A two-sample Mendelian randomization study
Source: Medicine (Baltimore). 2023 Sep 22;102(38):e35037. doi: 10.1097/MD.0000000000035037 (PMC10519452; doi:10.1097/MD.0000000000035037)
Supplement: Supplementary file 1 [file medi-102-e35037-s001.docx]

Supplementary Table S1 Characteristics of data sources.

| Outcome | Year | PMID | Author | Sample size | Ancestry | |
| --- | --- | --- | --- | --- | --- | --- |
| ieu-a-1126 Breast cancer | 2017 | 29059683 | Michailidou K | 122977 cases and 105974 controls | | European |
| ieu-a-1127 ER+ Breast cancer | 2017 | 29059683 | Michailidou K | 69501 cases and 105974 controls | European | |
| ieu-a-1128 ER- Breast cancer | 2017 | 29059683 | Michailidou K | 21468 cases and 105974 controls | European | |
| ebi-a-GCST005838 stroke | 2018 | 29531354 | Malik R | 40585 cases and 406111 controls | European | |
| ebi-a-GCST006908 Ischemic stroke | 2018 | 29531354 | Malik R | 34217 cases and 406111 controls | European | |
| ebi-a-GCST005842 Ischemic stroke (cardioembolic) | 2018 | 29531354 | Malik R | 7193 cases and 406111 controls | European | |
| ebi-a-GCST005841 Ischemic stroke (small-vessel) | 2018 | 29531354 | Malik R | 5386 cases and 192662 controls | European | |
| ebi-a-GCST005840  Ischemic stroke (large artery atherosclerosis) | 2018 | 29531354 | Malik R | 4373 cases and 406111 controls | European | |
