# Supplementary material for: Investigating the association of breast cancer and stroke: A two-sample Mendelian randomization study
Source: Medicine (Baltimore). 2023 Sep 22;102(38):e35037. doi: 10.1097/MD.0000000000035037 (PMC10519452; doi:10.1097/MD.0000000000035037)
Supplement: Supplementary file 2 [file medi-102-e35037-s002.docx]

**Supplementary Table S2 Characteristics of the instrumental variables for breast cancer and their relationship with stroke.**

| Exposure | SNP | Effect allele | Other allele | Exposure effect |  |  |
| --- | --- | --- | --- | --- | --- | --- |
|  |  |  |  | β | SE | P |
| Breast cancer | rs2506885 | T | A | -0.0629 | 0.0068 | 2.24E-20 |
|  | rs2992756 | C | T | -0.0506 | 0.0063 | 9.61E-16 |
|  | rs4233486 | T | C | 0.0396 | 0.0069 | 9.52E-09 |
|  | rs1707302 | G | A | 0.0364 | 0.0066 | 3.48E-08 |
|  | rs17426269 | A | G | 0.0487 | 0.0086 | 1.49E-08 |
|  | rs11583393 | A | C | -0.0396 | 0.0072 | 3.80E-08 |
|  | rs115532124 | A | G | -0.0615 | 0.0094 | 6.05E-11 |
|  | rs11249433 | G | A | 0.0988 | 0.0065 | 3.54E-52 |
|  | rs1778523 | G | C | -0.0384 | 0.0066 | 5.95E-09 |
|  | rs11205303 | C | T | 0.0497 | 0.0064 | 8.12E-15 |
|  | rs4971059 | A | G | 0.0424 | 0.0064 | 3.47E-11 |
|  | rs35383942 | T | C | 0.101 | 0.0139 | 3.70E-13 |
|  | rs7550984 | A | G | 0.0527 | 0.0093 | 1.46E-08 |
|  | rs11117758 | A | G | -0.0449 | 0.0076 | 3.47E-09 |
|  | rs72755295 | G | A | 0.1376 | 0.0179 | 1.50E-14 |
|  | rs113577745 | G | C | 0.064 | 0.0102 | 3.51E-10 |
|  | rs11684853 | T | G | -0.0442 | 0.0062 | 1.01E-12 |
|  | rs6725517 | G | A | -0.0468 | 0.0067 | 2.85E-12 |
|  | rs11903787 | A | G | -0.0451 | 0.0076 | 2.95E-09 |
|  | rs73949122 | G | C | -0.0593 | 0.0084 | 1.67E-12 |
|  | rs4848599 | C | T | 0.0933 | 0.01 | 1.06E-20 |
|  | rs2016394 | A | G | -0.0425 | 0.0062 | 7.14E-12 |
|  | rs3769821 | T | C | -0.0565 | 0.0065 | 3.55E-18 |
|  | rs4442975 | T | G | -0.1274 | 0.0061 | 7.29E-97 |
|  | rs11693806 | G | C | -0.0739 | 0.007 | 4.71E-26 |
|  | rs6436017 | G | A | 0.0399 | 0.0063 | 2.40E-10 |
|  | rs12479355 | G | A | -0.0426 | 0.0076 | 2.08E-08 |
|  | rs6762558 | G | A | 0.056 | 0.0063 | 6.17E-19 |
|  | rs7626742 | T | G | 0.1046 | 0.0062 | 7.36E-64 |
|  | rs12487185 | G | A | 0.045 | 0.0066 | 9.22E-12 |
|  | rs56387622 | C | T | -0.0942 | 0.0106 | 6.29E-19 |
|  | rs3821902 | G | T | 0.0623 | 0.0089 | 2.56E-12 |
|  | rs13066793 | G | A | -0.0685 | 0.0112 | 9.59E-10 |
|  | rs9833888 | T | G | 0.0457 | 0.0074 | 6.59E-10 |
|  | rs7650602 | C | T | 0.0489 | 0.0063 | 8.37E-15 |
|  | rs73031214 | T | C | 0.0474 | 0.0074 | 1.50E-10 |
|  | rs6815814 | C | A | 0.052 | 0.0072 | 5.11E-13 |
|  | rs28869016 | G | A | 0.0372 | 0.0063 | 3.53E-09 |
|  | rs10022462 | T | C | 0.0375 | 0.0062 | 1.46E-09 |
|  | rs62331151 | G | A | 0.0488 | 0.0073 | 2.31E-11 |
|  | rs77528541 | T | G | -0.0583 | 0.0096 | 1.26E-09 |
|  | rs9884717 | G | A | -0.1029 | 0.0097 | 2.73E-26 |
|  | rs12513872 | A | G | 0.0413 | 0.0073 | 1.54E-08 |
|  | rs10054203 | C | G | 0.0383 | 0.0065 | 3.81E-09 |
|  | rs2853669 | G | A | -0.0651 | 0.0069 | 3.92E-21 |
|  | rs4702131 | C | T | -0.0424 | 0.0062 | 7.99E-12 |
|  | rs12519859 | A | G | 0.0365 | 0.0063 | 6.89E-09 |
|  | rs10941679 | G | A | 0.1278 | 0.0071 | 1.95E-72 |
|  | rs59957907 | G | A | 0.1734 | 0.0082 | 2.98E-99 |
|  | rs1498608 | A | T | -0.0671 | 0.0107 | 3.59E-10 |
|  | rs2407064 | A | C | 0.0515 | 0.0072 | 8.50E-13 |
|  | rs12110303 | T | G | -0.0606 | 0.0089 | 9.83E-12 |
|  | rs6882649 | T | G | 0.0388 | 0.0066 | 4.13E-09 |
|  | rs56083805 | C | T | -0.044 | 0.0076 | 7.06E-09 |
|  | rs11135046 | T | G | -0.0717 | 0.0062 | 6.23E-31 |
|  | rs4562056 | T | G | 0.0416 | 0.0067 | 5.33E-10 |
|  | rs418053 | C | G | -0.0465 | 0.0063 | 1.57E-13 |
|  | rs71557345 | A | G | -0.0806 | 0.0129 | 4.16E-10 |
|  | rs12207986 | A | G | 0.0375 | 0.0062 | 1.46E-09 |
|  | rs9361840 | G | A | 0.0541 | 0.0072 | 5.74E-14 |
|  | rs6569648 | T | C | 0.0512 | 0.0073 | 2.32E-12 |
|  | rs60954078 | G | A | 0.1817 | 0.0117 | 2.18E-54 |
|  | rs76956704 | T | C | 0.1352 | 0.0182 | 1.10E-13 |
|  | rs2747651 | C | G | 0.0662 | 0.0062 | 1.30E-26 |
|  | rs7971 | G | A | -0.0365 | 0.0065 | 1.96E-08 |
|  | rs17156577 | C | T | 0.0578 | 0.0098 | 3.68E-09 |
|  | rs17268829 | C | T | 0.0495 | 0.0068 | 3.35E-13 |
|  | rs71559437 | A | G | -0.0686 | 0.0099 | 4.23E-12 |
|  | rs68056147 | A | G | 0.0501 | 0.0069 | 3.85E-13 |
|  | rs11977670 | A | G | 0.0522 | 0.0063 | 1.17E-16 |
|  | rs62485509 | T | G | -0.0545 | 0.0079 | 5.25E-12 |
|  | rs9693444 | C | A | -0.0626 | 0.0066 | 2.43E-21 |
|  | rs4286946 | G | C | -0.0799 | 0.0083 | 6.18E-22 |
|  | rs1397734 | G | A | 0.0773 | 0.0082 | 4.23E-21 |
|  | rs72658071 | T | A | 0.1182 | 0.0107 | 2.27E-28 |
|  | rs12546444 | T | A | -0.0717 | 0.011 | 7.12E-11 |
|  | rs13267382 | G | A | -0.0437 | 0.0065 | 1.78E-11 |
|  | rs12545624 | G | A | 0.0609 | 0.011 | 3.09E-08 |
|  | rs10096351 | G | A | 0.1055 | 0.0062 | 6.24E-65 |
|  | rs1121948 | G | A | 0.0575 | 0.0077 | 8.17E-14 |
|  | rs3217992 | T | C | -0.0581 | 0.0064 | 1.10E-19 |
|  | rs10978911 | C | G | 0.0839 | 0.0091 | 2.98E-20 |
|  | rs548980 | T | C | 0.0993 | 0.0064 | 2.72E-54 |
|  | rs35910339 | G | C | 0.0462 | 0.0067 | 5.37E-12 |
|  | rs10760444 | A | G | -0.0358 | 0.0062 | 7.73E-09 |
|  | rs532436 | A | G | 0.0431 | 0.0078 | 3.28E-08 |
|  | rs67801543 | T | C | 0.0568 | 0.0094 | 1.52E-09 |
|  | rs7072776 | G | A | -0.0618 | 0.0068 | 1.01E-19 |
|  | rs66572733 | G | C | 0.095 | 0.0162 | 4.51E-09 |
|  | rs10995201 | G | A | -0.1317 | 0.0087 | 9.10E-52 |
|  | rs1268974 | G | A | -0.0793 | 0.0063 | 2.48E-36 |
|  | rs7904249 | A | G | 0.0764 | 0.0084 | 9.44E-20 |
|  | rs10885405 | T | C | 0.0465 | 0.0062 | 6.38E-14 |
|  | rs12250948 | C | T | -0.0576 | 0.0074 | 7.04E-15 |
|  | rs9420318 | A | G | -0.0464 | 0.0066 | 2.06E-12 |
|  | rs2912780 | T | C | -0.2373 | 0.0063 | 0 |
|  | rs6597981 | G | A | 0.0439 | 0.0062 | 1.43E-12 |
|  | rs1973765 | C | T | -0.0817 | 0.0064 | 2.55E-37 |
|  | rs3903072 | T | G | -0.0434 | 0.0062 | 2.56E-12 |
|  | rs117737783 | A | G | 0.0747 | 0.0127 | 4.06E-09 |
|  | rs57212245 | A | G | -0.0652 | 0.0119 | 4.28E-08 |
|  | rs78540526 | T | C | 0.2758 | 0.0113 | ####### |
|  | rs11822830 | G | A | 0.0483 | 0.0063 | 1.77E-14 |
|  | rs11571833 | T | A | 0.2727 | 0.0346 | 3.23E-15 |
|  | rs6562760 | G | A | 0.0443 | 0.0073 | 1.29E-09 |
|  | rs7149262 | A | C | -0.0724 | 0.0076 | 1.63E-21 |
|  | rs2253012 | T | C | 0.0433 | 0.0065 | 2.71E-11 |
|  | rs72725173 | A | G | 0.0559 | 0.0085 | 4.82E-11 |
|  | rs11624333 | C | T | -0.0968 | 0.0071 | 2.52E-42 |
|  | rs941764 | G | A | 0.0463 | 0.0065 | 1.06E-12 |
|  | rs78440108 | T | C | -0.0595 | 0.0084 | 1.41E-12 |
|  | rs4983544 | G | T | 0.0353 | 0.0063 | 2.10E-08 |
|  | rs12594752 | T | C | -0.0729 | 0.0092 | 2.30E-15 |
|  | rs35850695 | A | G | 0.2097 | 0.0069 | ####### |
|  | rs11642015 | T | C | -0.0624 | 0.0063 | 3.97E-23 |
|  | rs7184573 | A | G | -0.047 | 0.0065 | 4.80E-13 |
|  | rs13329835 | G | A | 0.0786 | 0.0073 | 4.92E-27 |
|  | rs4496150 | A | C | -0.0416 | 0.0072 | 7.57E-09 |
|  | rs62070645 | A | C | -0.0439 | 0.007 | 3.58E-10 |
|  | rs78381082 | G | A | -0.0611 | 0.0089 | 6.64E-12 |
|  | rs1812715 | G | A | 0.0748 | 0.0067 | 6.11E-29 |
|  | rs8082452 | G | T | -0.0418 | 0.0065 | 1.27E-10 |
|  | rs521667 | C | A | 0.0475 | 0.0066 | 6.16E-13 |
|  | rs170801 | A | C | -0.0591 | 0.007 | 3.10E-17 |
|  | rs72933507 | C | T | -0.1028 | 0.0149 | 5.22E-12 |
|  | rs9954058 | C | G | -0.0891 | 0.0122 | 2.81E-13 |
|  | rs9952980 | C | T | -0.0478 | 0.0065 | 1.93E-13 |
|  | rs56013747 | C | A | -0.0497 | 0.009 | 3.35E-08 |
|  | rs8105994 | C | T | -0.0722 | 0.0065 | 1.15E-28 |
|  | rs2905427 | T | G | -0.0445 | 0.0065 | 7.59E-12 |
|  | rs56681946 | C | T | 0.0607 | 0.0066 | 3.68E-20 |
|  | rs11672660 | T | C | 0.0472 | 0.008 | 3.64E-09 |
|  | rs16991615 | A | G | 0.0758 | 0.0126 | 1.79E-09 |
|  | rs6122906 | G | A | 0.0507 | 0.008 | 2.34E-10 |
|  | rs2403907 | A | C | -0.0796 | 0.0067 | 1.49E-32 |
|  | rs62237573 | T | C | 0.4256 | 0.034 | 5.98E-36 |
|  | rs5997389 | A | G | 0.0791 | 0.0108 | 2.41E-13 |
|  | rs4820318 | A | G | -0.0475 | 0.0064 | 1.15E-13 |
|  | rs6001974 | G | A | 0.1208 | 0.0098 | 6.52E-35 |
|  | rs28512361 | A | G | 0.0611 | 0.0109 | 2.08E-08 |
| ER-positive breast cancer | rs17035305 | A | C | -0.0436 | 0.0079 | 3.41E-08 |
|  | rs2992756 | C | T | -0.0608 | 0.0076 | 1.24E-15 |
|  | rs1707302 | G | A | 0.0466 | 0.0078 | 2.31E-09 |
|  | rs17426269 | A | G | 0.0576 | 0.0103 | 2.24E-08 |
|  | rs35273427 | C | T | 0.0937 | 0.016 | 4.73E-09 |
|  | rs115532124 | A | G | -0.0753 | 0.011 | 7.62E-12 |
|  | rs11249433 | G | A | 0.123 | 0.0077 | 1.94E-57 |
|  | rs35353761 | G | A | -0.06 | 0.01 | 1.97E-09 |
|  | rs11205277 | G | A | 0.0562 | 0.0075 | 6.71E-14 |
|  | rs4971059 | A | G | 0.0519 | 0.0077 | 1.58E-11 |
|  | rs35383942 | T | C | 0.0918 | 0.0164 | 2.17E-08 |
|  | rs11117758 | A | G | -0.05 | 0.0091 | 3.92E-08 |
|  | rs72755295 | G | A | 0.1481 | 0.0208 | 1.08E-12 |
|  | rs4076654 | T | A | -0.0595 | 0.0079 | 5.01E-14 |
|  | rs12711947 | C | T | 0.0864 | 0.0124 | 3.22E-12 |
|  | rs2016394 | A | G | -0.0603 | 0.0074 | 3.68E-16 |
|  | rs3769821 | T | C | -0.0545 | 0.0078 | 2.80E-12 |
|  | rs4442975 | T | G | -0.1482 | 0.0073 | 1.25E-91 |
|  | rs11693806 | G | C | -0.0722 | 0.0083 | 3.35E-18 |
|  | rs6787391 | T | C | 0.062 | 0.0076 | 3.41E-16 |
|  | rs490706 | A | G | 0.1148 | 0.0074 | 2.81E-54 |
|  | rs17838698 | T | C | 0.0624 | 0.008 | 6.19E-15 |
|  | rs56387622 | C | T | -0.0991 | 0.0127 | 6.04E-15 |
|  | rs3821902 | G | T | 0.0589 | 0.0106 | 2.75E-08 |
|  | rs62247166 | T | C | -0.0406 | 0.0074 | 4.10E-08 |
|  | rs4361315 | A | G | -0.0547 | 0.0087 | 3.23E-10 |
|  | rs7650602 | C | T | 0.0571 | 0.0075 | 2.67E-14 |
|  | rs9868605 | C | T | 0.0516 | 0.0088 | 4.53E-09 |
|  | rs6815814 | C | A | 0.055 | 0.0086 | 1.60E-10 |
|  | rs17617028 | A | G | 0.0569 | 0.0087 | 6.14E-11 |
|  | rs985261 | T | C | 0.1339 | 0.0118 | 7.64E-30 |
|  | rs2735846 | G | C | -0.0445 | 0.0075 | 2.97E-09 |
|  | rs17611291 | C | G | -0.041 | 0.0074 | 3.02E-08 |
|  | rs10941679 | G | A | 0.1563 | 0.0084 | 2.81E-77 |
|  | rs889310 | T | C | 0.0462 | 0.0078 | 3.16E-09 |
|  | rs62355901 | C | T | 0.2027 | 0.0097 | 5.71E-97 |
|  | rs4703870 | A | G | 0.0613 | 0.0088 | 3.26E-12 |
|  | rs12110303 | T | G | -0.074 | 0.0107 | 4.65E-12 |
|  | rs56083805 | C | T | -0.0595 | 0.0091 | 6.22E-11 |
|  | rs1432679 | T | C | -0.0695 | 0.0074 | 5.89E-21 |
|  | rs7729559 | T | C | 0.057 | 0.0088 | 9.34E-11 |
|  | rs418053 | C | G | -0.0524 | 0.0075 | 2.81E-12 |
|  | rs34196306 | C | G | -0.0889 | 0.015 | 3.09E-09 |
|  | rs1361549 | A | G | -0.0451 | 0.0075 | 1.82E-09 |
|  | rs60954078 | G | A | 0.1392 | 0.014 | 2.71E-23 |
|  | rs6904031 | T | A | 0.1107 | 0.0148 | 7.45E-14 |
|  | rs17268829 | C | T | 0.0517 | 0.0082 | 2.88E-10 |
|  | rs7796917 | A | G | -0.0794 | 0.0119 | 2.52E-11 |
|  | rs12703654 | A | C | 0.0587 | 0.0077 | 2.47E-14 |
|  | rs62485509 | T | G | -0.053 | 0.0094 | 1.72E-08 |
|  | rs310302 | A | G | 0.0444 | 0.0075 | 3.22E-09 |
|  | rs7463114 | C | T | -0.0684 | 0.0078 | 1.80E-18 |
|  | rs4286946 | G | C | -0.0748 | 0.0099 | 4.17E-14 |
|  | rs6472903 | T | G | 0.0784 | 0.0099 | 2.39E-15 |
|  | rs72658071 | T | A | 0.1187 | 0.0128 | 1.80E-20 |
|  | rs16867692 | G | A | 0.0576 | 0.0096 | 1.97E-09 |
|  | rs2957449 | C | T | 0.0475 | 0.0076 | 4.10E-10 |
|  | rs13267382 | G | A | -0.0427 | 0.0077 | 2.93E-08 |
|  | rs12550713 | G | C | 0.1093 | 0.0075 | 4.15E-48 |
|  | rs7017073 | C | T | 0.0628 | 0.0089 | 1.71E-12 |
|  | rs3217992 | T | C | -0.0576 | 0.0076 | 3.48E-14 |
|  | rs10978911 | C | G | 0.0978 | 0.0107 | 6.23E-20 |
|  | rs7862747 | A | C | 0.1237 | 0.0077 | 4.49E-58 |
|  | rs811458 | C | T | 0.0511 | 0.008 | 1.69E-10 |
|  | rs10828248 | G | A | 0.0726 | 0.0077 | 4.16E-21 |
|  | rs2009607 | T | A | 0.1165 | 0.0202 | 8.05E-09 |
|  | rs10995201 | G | A | -0.1379 | 0.0105 | 2.12E-39 |
|  | rs946008 | T | C | -0.0861 | 0.0077 | 5.01E-29 |
|  | rs4980029 | G | A | 0.0759 | 0.0098 | 9.57E-15 |
|  | rs12250948 | C | T | -0.0644 | 0.0089 | 4.62E-13 |
|  | rs9420318 | A | G | -0.0589 | 0.0079 | 8.94E-14 |
|  | rs11200014 | A | G | 0.2853 | 0.0075 | 0 |
|  | rs2461201 | T | C | 0.0473 | 0.0078 | 1.33E-09 |
|  | rs688601 | A | T | -0.0832 | 0.0074 | 2.50E-29 |
|  | rs10896047 | A | G | -0.0493 | 0.0074 | 2.70E-11 |
|  | rs78540526 | T | C | 0.3277 | 0.0132 | ####### |
|  | rs11822830 | G | A | 0.0445 | 0.0075 | 2.97E-09 |
|  | rs11571818 | C | T | 0.239 | 0.0397 | 1.74E-09 |
|  | rs7149262 | A | C | -0.0804 | 0.0091 | 1.00E-18 |
|  | rs2253012 | T | C | 0.0511 | 0.0078 | 5.70E-11 |
|  | rs28649231 | A | G | 0.0651 | 0.0095 | 7.25E-12 |
|  | rs11624164 | T | C | -0.1037 | 0.0086 | 1.76E-33 |
|  | rs7153397 | T | C | 0.0536 | 0.0081 | 3.66E-11 |
|  | rs80046058 | T | A | -0.0498 | 0.0088 | 1.52E-08 |
|  | rs6938 | G | C | 0.0446 | 0.0081 | 3.67E-08 |
|  | rs12594752 | T | C | -0.0754 | 0.0109 | 4.60E-12 |
|  | rs4784227 | T | C | 0.2296 | 0.0084 | ####### |
|  | rs62048402 | A | G | -0.0593 | 0.0075 | 2.64E-15 |
|  | rs7500067 | G | A | 0.0819 | 0.0086 | 1.68E-21 |
|  | rs7223535 | A | G | -0.0463 | 0.0084 | 3.55E-08 |
|  | rs1812715 | G | A | 0.0908 | 0.0081 | 3.65E-29 |
|  | rs521667 | C | A | 0.0482 | 0.0078 | 6.43E-10 |
|  | rs170801 | A | C | -0.0794 | 0.0084 | 3.31E-21 |
|  | rs9954058 | C | G | -0.1057 | 0.0147 | 6.46E-13 |
|  | rs9952980 | C | T | -0.0549 | 0.0078 | 1.94E-12 |
|  | rs8105994 | C | T | -0.0768 | 0.0078 | 7.12E-23 |
|  | rs1685191 | T | C | -0.0635 | 0.0077 | 1.63E-16 |
|  | rs2238691 | A | G | 0.0557 | 0.0095 | 4.54E-09 |
|  | rs12481286 | T | G | 0.0507 | 0.0091 | 2.53E-08 |
|  | rs6062356 | G | T | 0.0564 | 0.0103 | 4.36E-08 |
|  | rs7280197 | C | T | 0.0461 | 0.0082 | 1.89E-08 |
|  | rs2403907 | A | C | -0.0964 | 0.008 | 1.94E-33 |
|  | rs62237573 | T | C | 0.4396 | 0.0386 | 4.77E-30 |
|  | rs5997389 | A | G | 0.0882 | 0.0128 | 5.55E-12 |
|  | rs4820318 | A | G | -0.0632 | 0.0076 | 9.11E-17 |
|  | rs6001982 | A | C | 0.1109 | 0.0117 | 2.58E-21 |
| ER-negative breast cancer | rs616402 | T | C | -0.1172 | 0.0124 | 3.34E-21 |
|  | rs12129456 | G | T | -0.0835 | 0.0118 | 1.48E-12 |
|  | rs2169137 | C | G | -0.128 | 0.0128 | 1.52E-23 |
|  | rs34697209 | G | A | -0.0673 | 0.0122 | 3.46E-08 |
|  | rs12472404 | C | G | -0.0879 | 0.0137 | 1.40E-10 |
|  | rs4528762 | C | A | -0.1213 | 0.0147 | 1.56E-16 |
|  | rs10179592 | C | T | 0.1156 | 0.0193 | 2.10E-09 |
|  | rs3769823 | G | A | -0.0684 | 0.0121 | 1.58E-08 |
|  | rs12990503 | G | C | -0.0734 | 0.0126 | 5.70E-09 |
|  | rs10069690 | T | C | 0.1613 | 0.013 | 2.37E-35 |
|  | rs2735846 | G | C | -0.0831 | 0.0117 | 1.22E-12 |
|  | rs7710996 | G | A | -0.0773 | 0.0132 | 4.74E-09 |
|  | rs2731830 | A | G | -0.2345 | 0.0419 | 2.19E-08 |
|  | rs1432679 | T | C | -0.073 | 0.0113 | 1.05E-10 |
|  | rs56309329 | T | A | 0.0734 | 0.0131 | 2.11E-08 |
|  | rs6569648 | T | C | 0.0741 | 0.0135 | 4.04E-08 |
|  | rs9397437 | A | G | 0.2733 | 0.0206 | 3.60E-40 |
|  | rs76956704 | T | C | 0.2193 | 0.0325 | 1.50E-11 |
|  | rs2747652 | C | T | 0.0991 | 0.0113 | 1.79E-18 |
|  | rs66823261 | C | T | 0.0836 | 0.0143 | 5.03E-09 |
|  | rs56687477 | A | G | -0.1114 | 0.016 | 3.34E-12 |
|  | rs189268208 | G | A | 0.1116 | 0.0196 | 1.24E-08 |
|  | rs10096351 | G | A | 0.07 | 0.0114 | 8.23E-10 |
|  | rs2263146 | T | C | 0.0797 | 0.0118 | 1.44E-11 |
|  | rs10995201 | G | A | -0.0963 | 0.0159 | 1.39E-09 |
|  | rs10885405 | T | C | 0.0716 | 0.0113 | 2.35E-10 |
|  | rs7129816 | A | G | -0.0629 | 0.0115 | 4.51E-08 |
|  | rs11571833 | T | A | 0.4346 | 0.0588 | 1.46E-13 |
|  | rs4143044 | G | T | 0.0833 | 0.0135 | 6.81E-10 |
|  | rs17828955 | C | T | -0.0797 | 0.013 | 8.75E-10 |
|  | rs11076805 | A | C | -0.0782 | 0.014 | 2.33E-08 |
|  | rs4784227 | T | C | 0.1368 | 0.0128 | 1.16E-26 |
|  | rs55872725 | T | C | -0.0784 | 0.0115 | 9.27E-12 |
|  | rs191981806 | C | T | 0.1963 | 0.036 | 4.96E-08 |
|  | rs56069439 | A | C | 0.155 | 0.0121 | 1.44E-37 |
|  | rs8104447 | C | T | 0.067 | 0.0115 | 5.67E-09 |
|  | rs183438976 | T | C | 0.1285 | 0.0186 | 4.89E-12 |
| Stroke | rs880315 | C | T | 0.0527 | 0.0084 | 3.52E-10 |
|  | rs10776752 | T | G | 0.0715 | 0.0128 | 2.32E-08 |
|  | rs11587860 | C | G | -0.0644 | 0.0086 | 6.97E-14 |
|  | rs2129983 | A | G | -0.0724 | 0.009 | 8.66E-16 |
|  | rs6825454 | C | T | 0.0518 | 0.0087 | 2.62E-09 |
|  | rs11957829 | G | A | -0.0654 | 0.0116 | 1.72E-08 |
|  | rs4959130 | A | G | 0.0779 | 0.0129 | 1.55E-09 |
|  | rs16896398 | T | A | 0.0477 | 0.0084 | 1.36E-08 |
|  | rs2107595 | A | G | 0.0727 | 0.0096 | 3.65E-14 |
|  | rs42039 | T | C | -0.0604 | 0.0105 | 8.80E-09 |
|  | rs7859727 | T | C | 0.0494 | 0.0079 | 4.02E-10 |
|  | rs11191833 | A | G | -0.0527 | 0.0082 | 1.30E-10 |
|  | rs10774624 | A | G | -0.0621 | 0.0091 | 8.84E-12 |
|  | rs35436 | T | C | -0.0462 | 0.0083 | 2.60E-08 |
|  | rs1535791 | C | T | 0.0606 | 0.0097 | 4.17E-10 |
|  | rs12445022 | A | G | 0.0574 | 0.0089 | 1.12E-10 |
|  | rs8103309 | C | T | -0.0501 | 0.0091 | 3.68E-08 |
| ischemic stroke | rs2758612 | C | T | -0.0653 | 0.0111 | 4.03E-09 |
|  | rs2634074 | A | T | -0.0941 | 0.0121 | 7.43E-15 |
|  | rs34311906 | C | T | 0.0649 | 0.0113 | 9.28E-09 |
|  | rs2066864 | A | G | 0.0634 | 0.0115 | 3.53E-08 |
|  | rs11242678 | T | C | 0.0723 | 0.0114 | 2.27E-10 |
|  | rs2107595 | A | G | 0.0882 | 0.0132 | 2.36E-11 |
|  | rs473238 | C | T | -0.0831 | 0.0147 | 1.58E-08 |
|  | rs3184504 | C | T | -0.0779 | 0.0101 | 1.23E-14 |
|  | rs4942561 | T | G | 0.0655 | 0.0116 | 1.64E-08 |
| Ischemic stroke (cardioembolic) | rs13143308 | G | T | -0.2798 | 0.0193 | 1.26E-47 |
|  | rs1906613 | A | G | 0.1019 | 0.0181 | 1.80E-08 |
|  | rs55884259 | A | G | -0.1096 | 0.0186 | 3.80E-09 |
|  | rs12932445 | C | T | 0.1839 | 0.0213 | 5.93E-18 |
| Ischemic stroke (large artery atherosclerosis) | rs12124533 | T | C | 0.1529 | 0.0268 | 1.16E-08 |
|  | rs17612742 | C | T | 0.176 | 0.0261 | 1.55E-11 |
|  | rs2107595 | A | G | 0.1871 | 0.0238 | 3.80E-15 |
|  | rs72985562 | G | T | 0.2449 | 0.0439 | 2.42E-08 |

SNP, single nucleotide polymorphism; SE, standard error.
