# Supplementary material for: Investigating the association of breast cancer and stroke: A two-sample Mendelian randomization study
Source: Medicine (Baltimore). 2023 Sep 22;102(38):e35037. doi: 10.1097/MD.0000000000035037 (PMC10519452; doi:10.1097/MD.0000000000035037)

Supplementary Figure S1. “leave-one-out” analysis plots for 27 causal associations with the breast cancer and stroke using the IVW test.

1. Breast cancer to Stroke


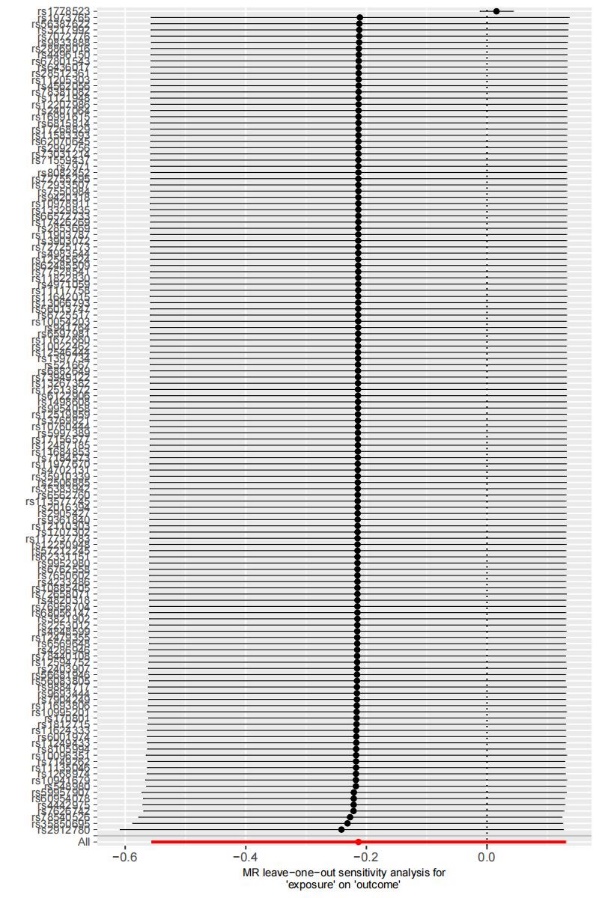


1. ER-positive breast cancer on Stroke


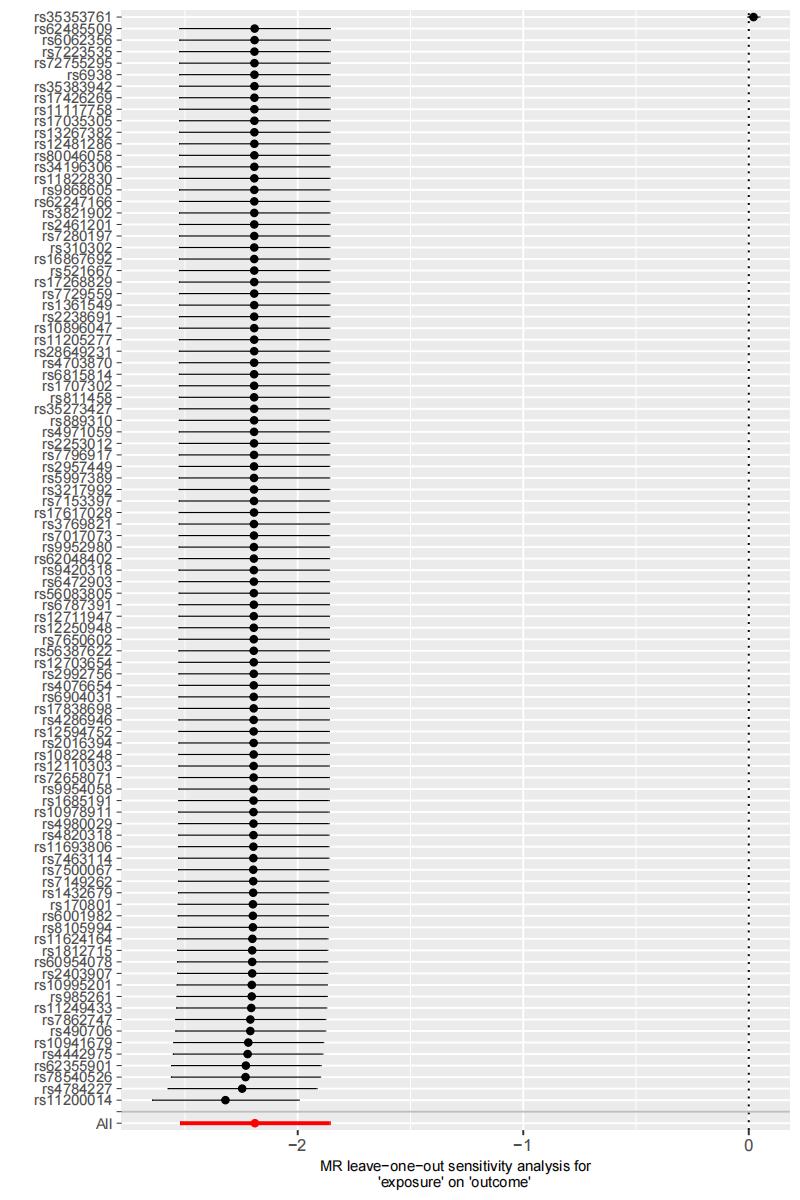


1. ER-negative breast cancer on Stroke


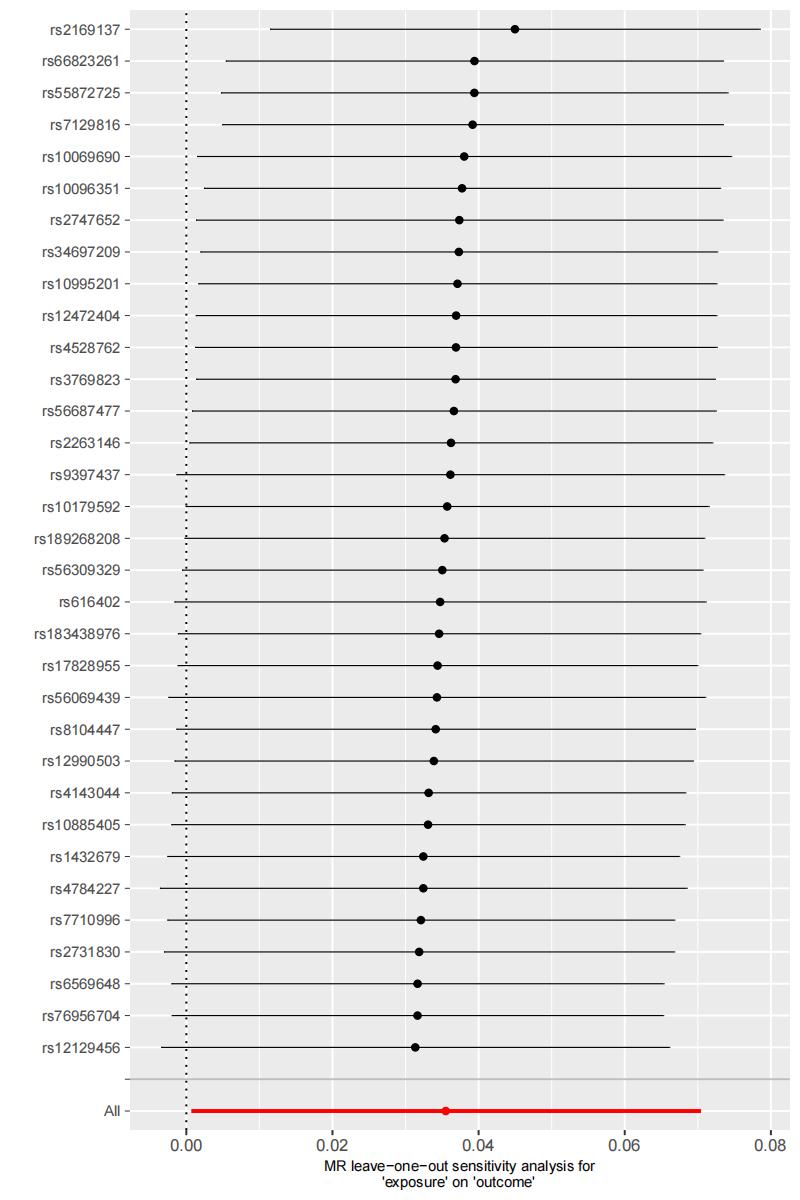


1. Breast cancer on Ischemic stroke


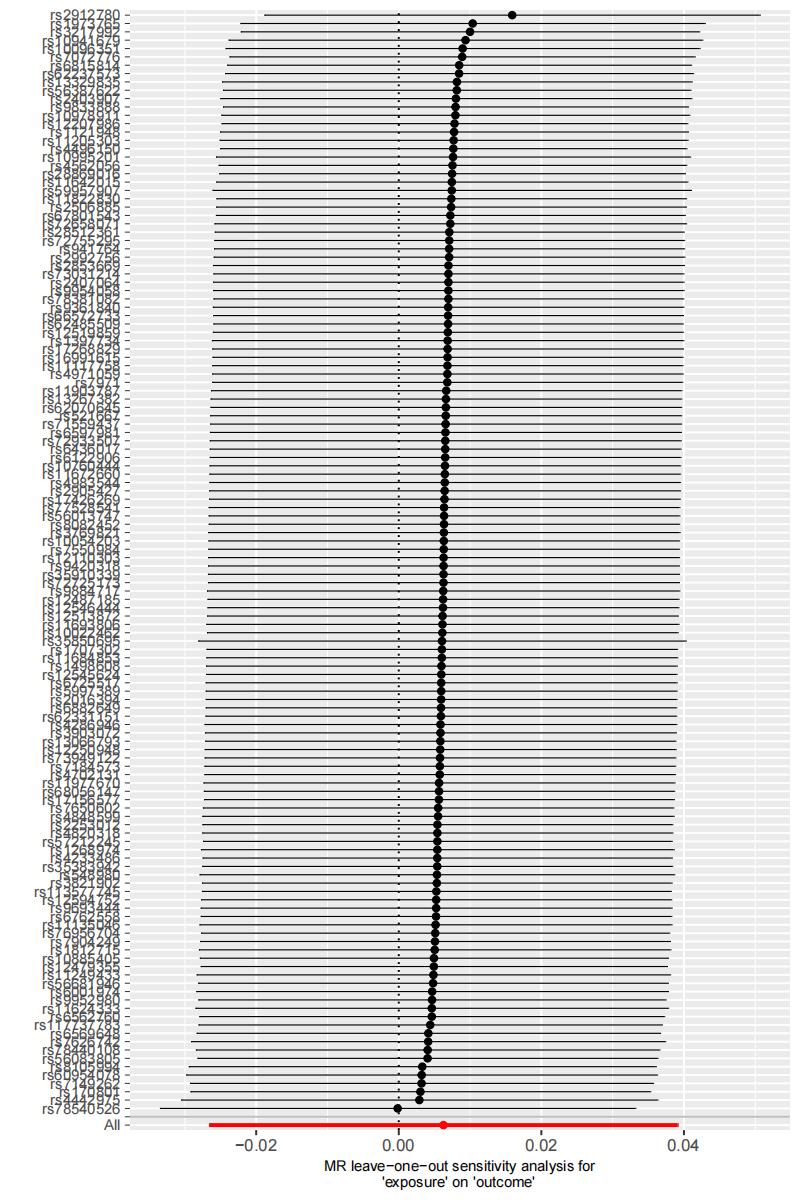


1. ER-positive breast cancer on Ischemic stroke


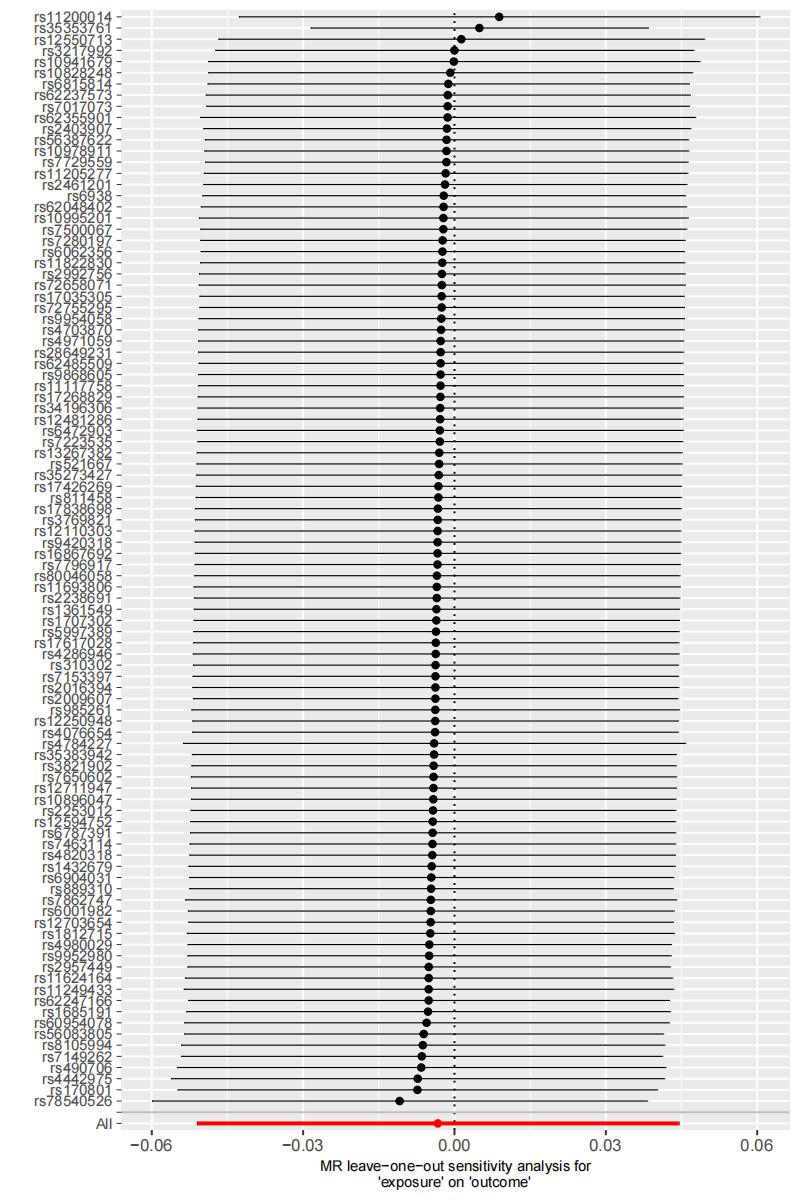


1. ER-negative breast cancer on Ischemic stroke


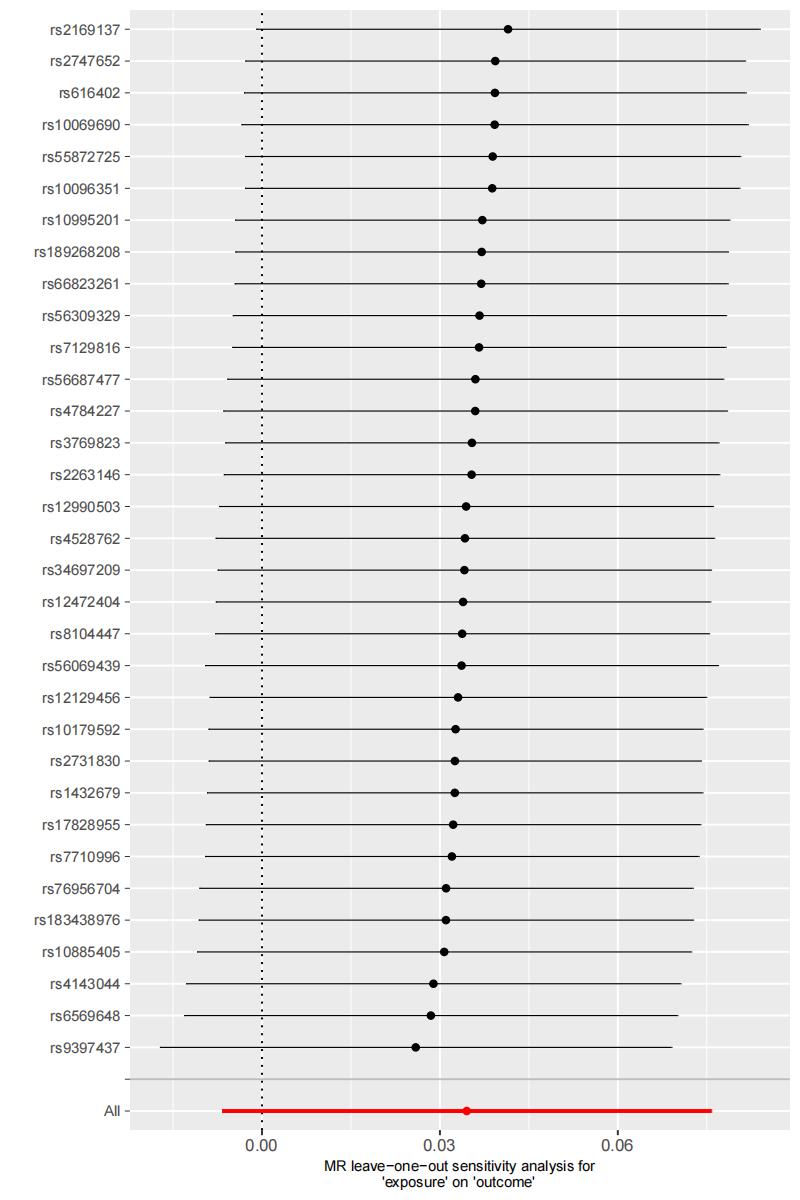


1. Breast cancer on Ischemic stroke (cardioembolic)


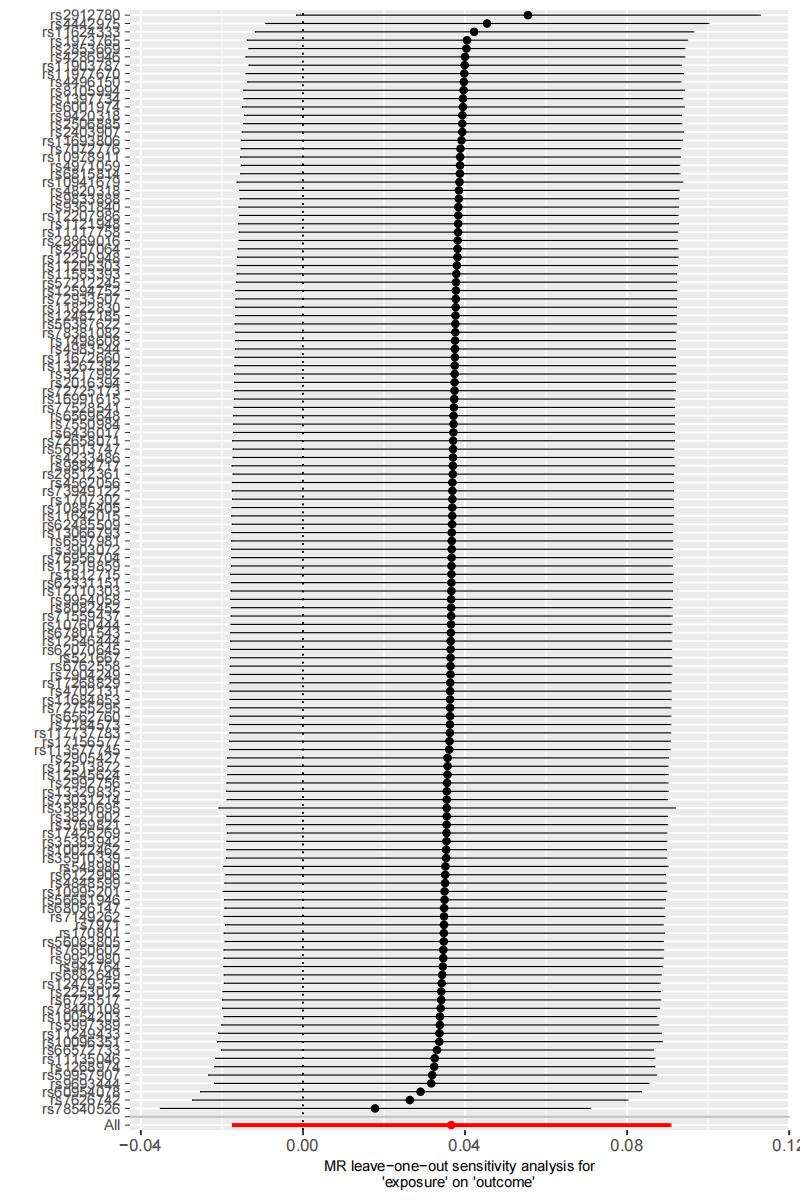


1. ER-positive breast cancer on Ischemic stroke (cardioembolic)


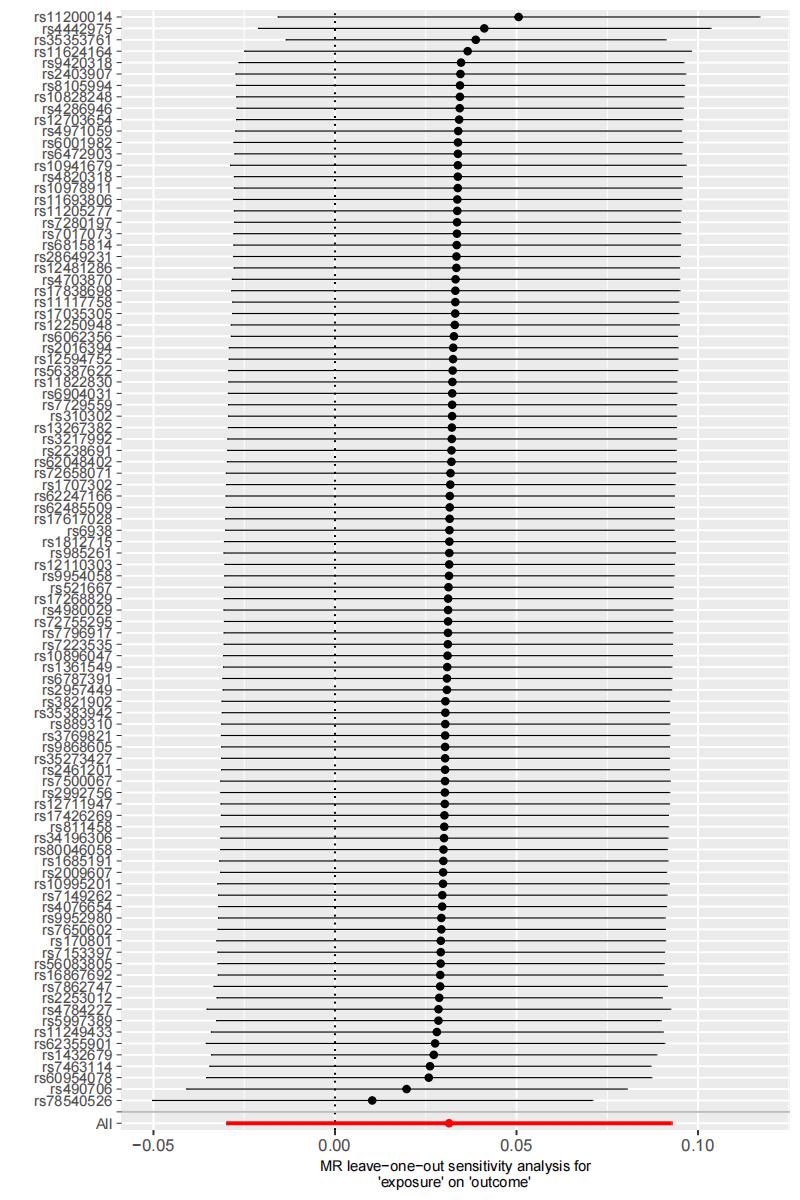


1. ER-negative breast cancer on Ischemic stroke (cardioembolic)


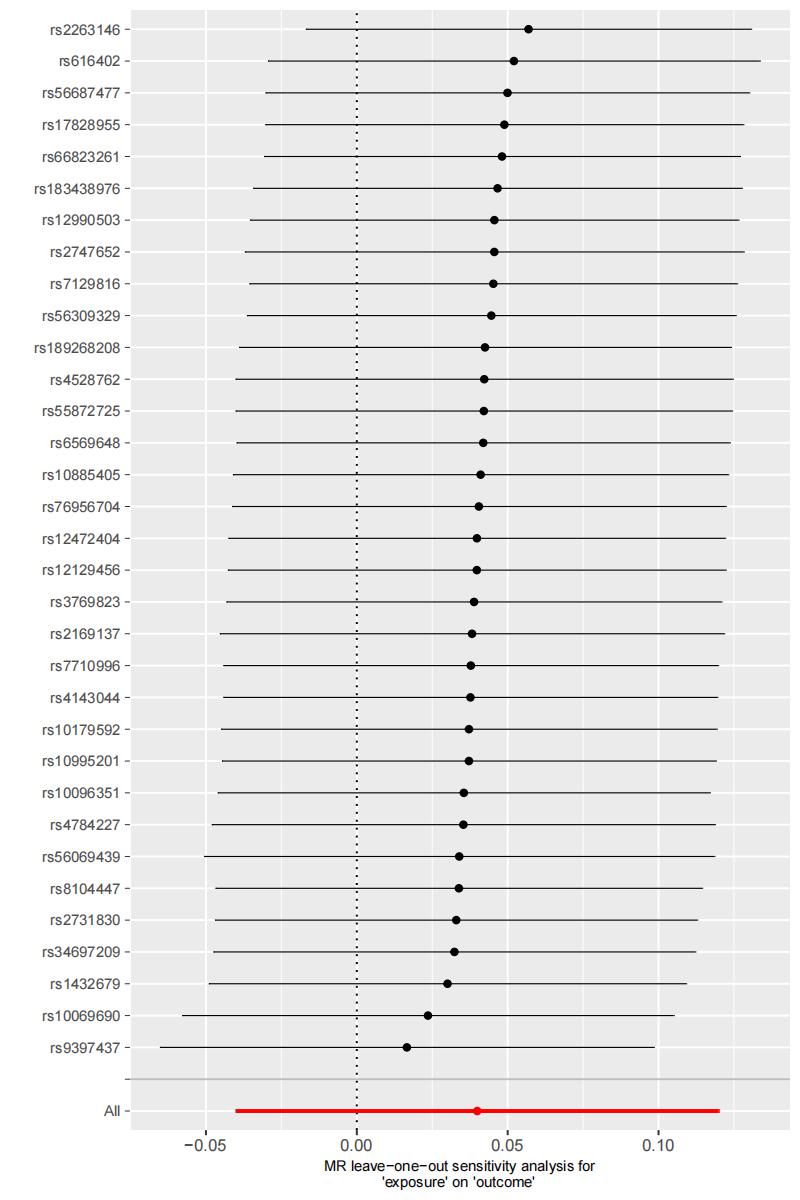


1. Breast cancer on Ischemic stroke (small-vessel)


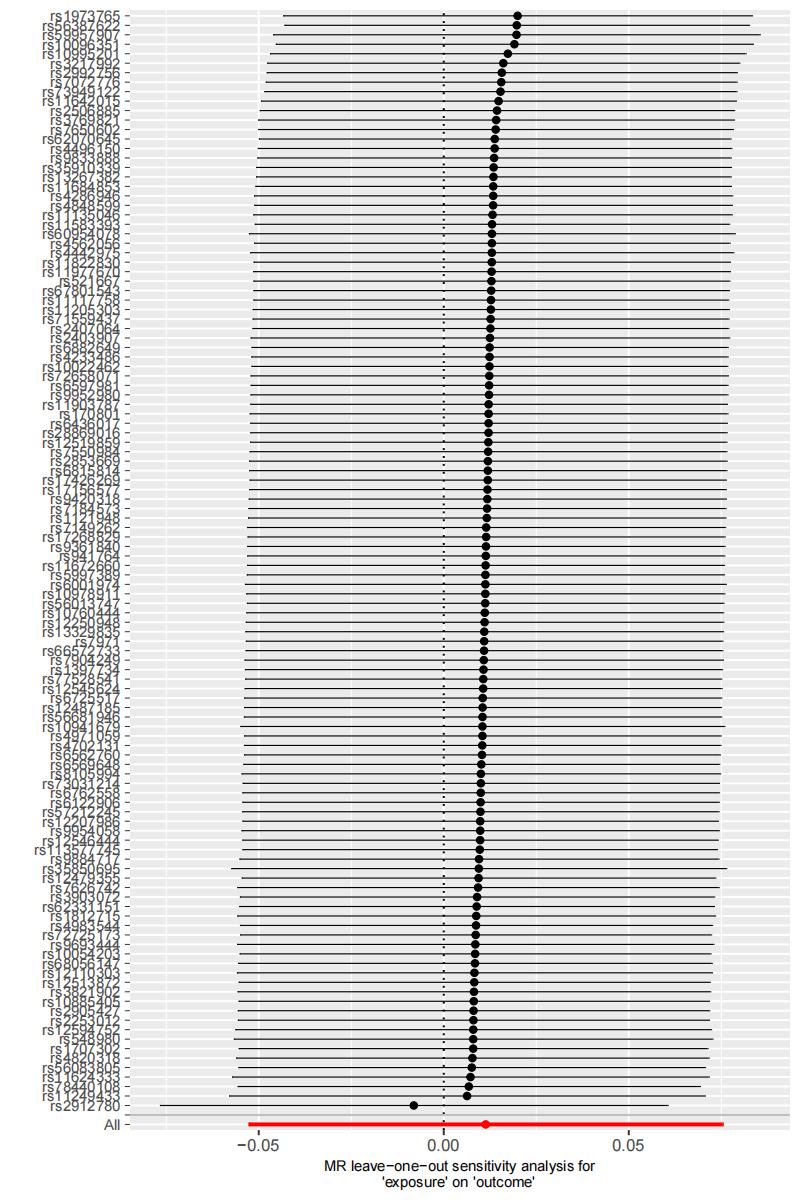


1. ER-positive breast cancer on Ischemic stroke (small-vessel)


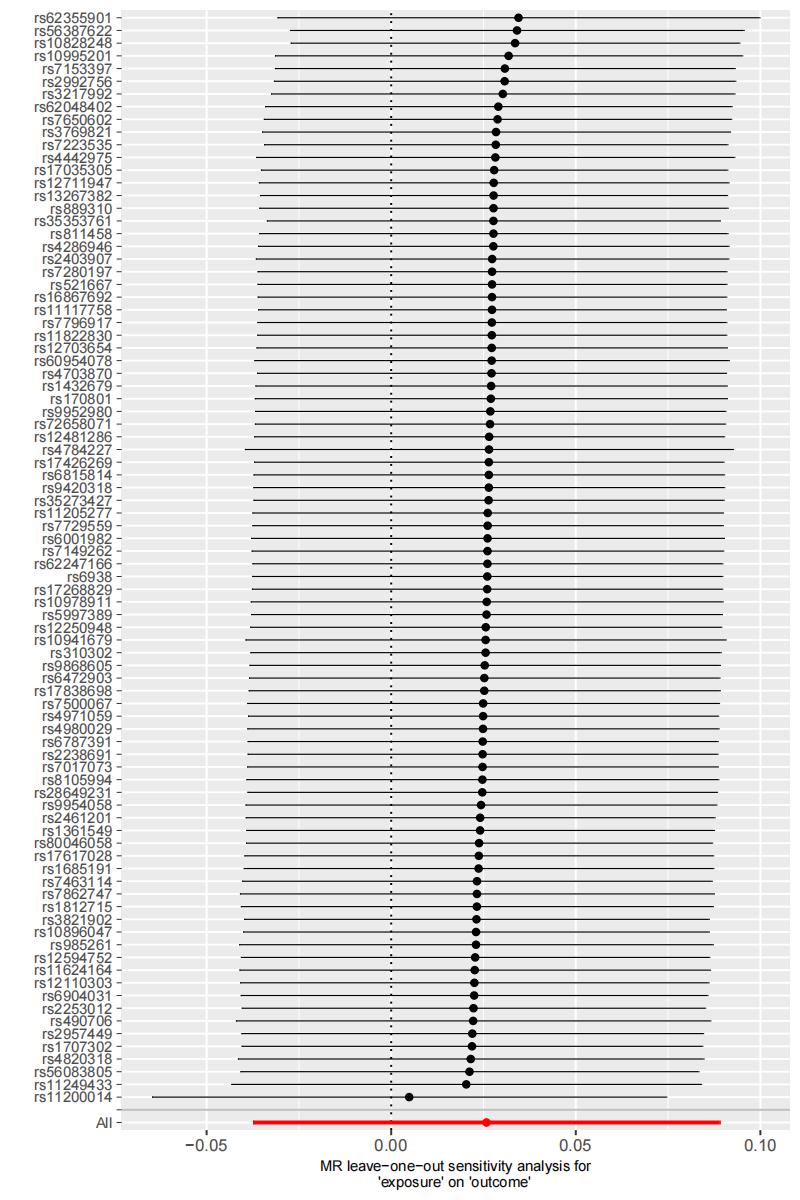


1. ER-negative breast cancer on Ischemic stroke (small-vessel)


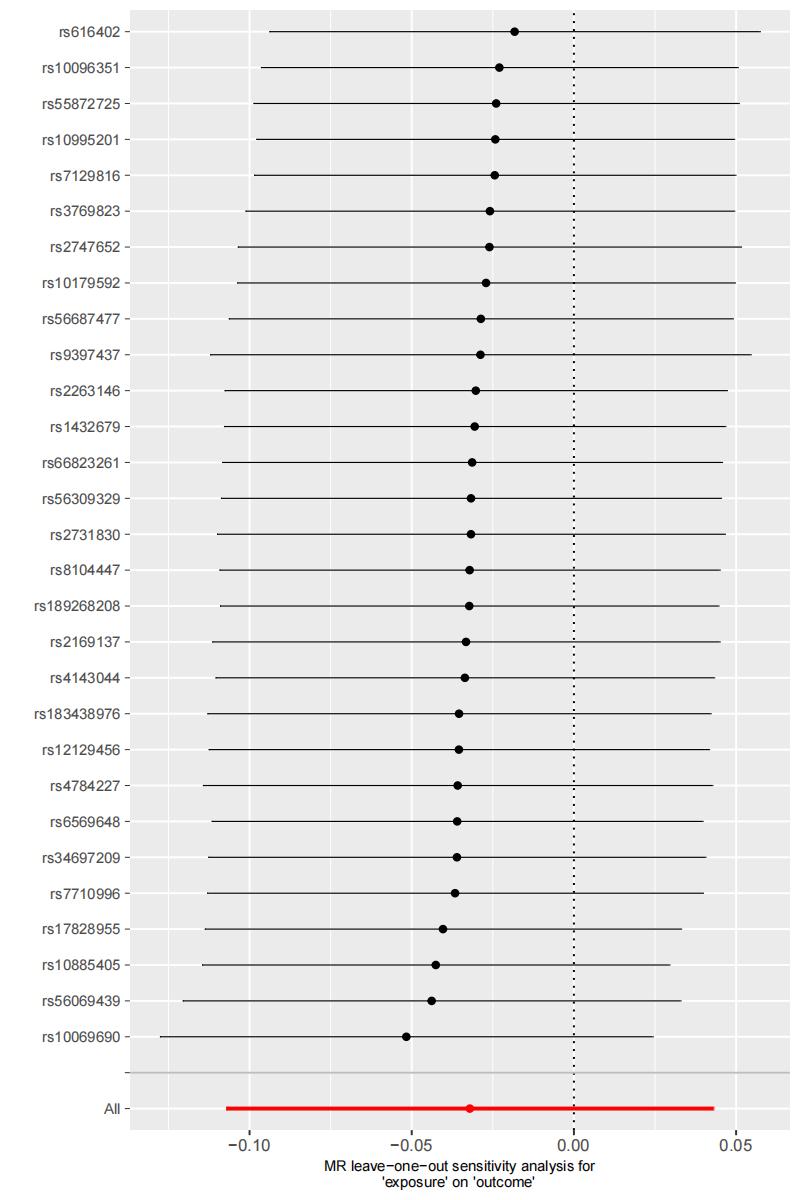


1. Breast cancer on Ischemic stroke (large artery atherosclerosis)


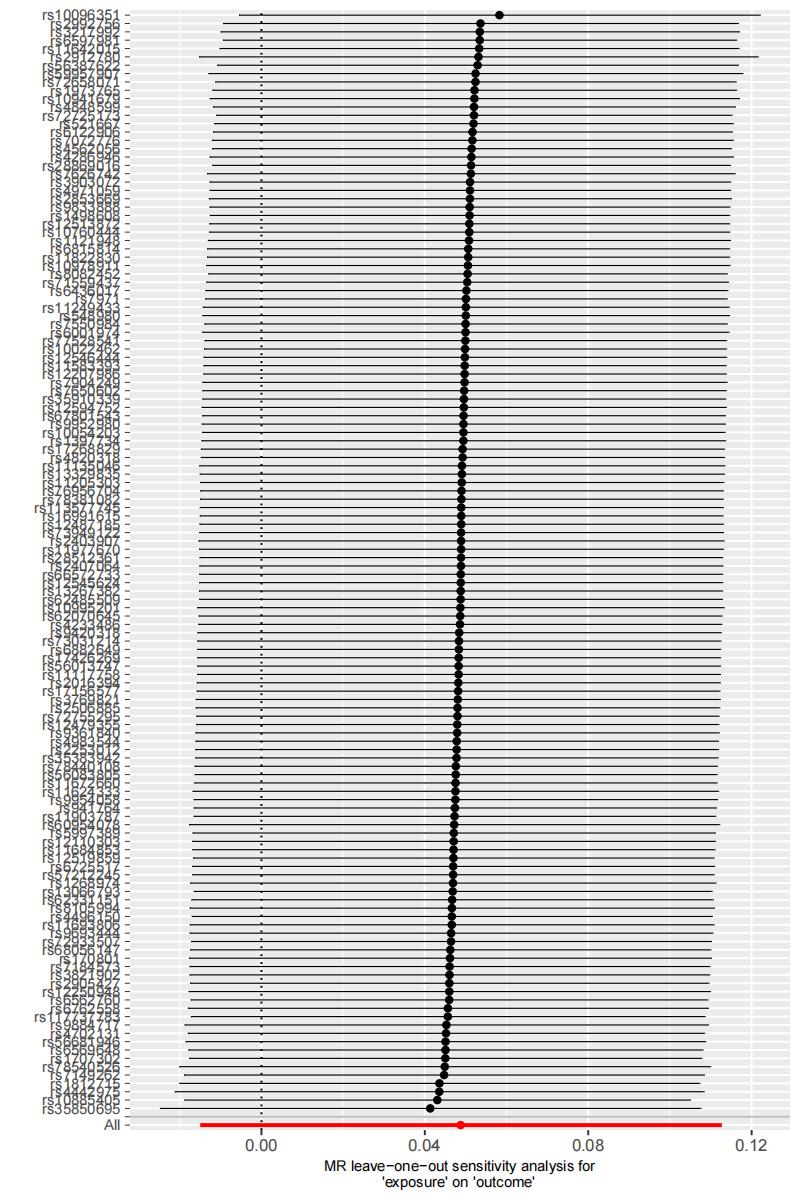


1. ER-positive breast cancer on Ischemic stroke (large artery atherosclerosis)


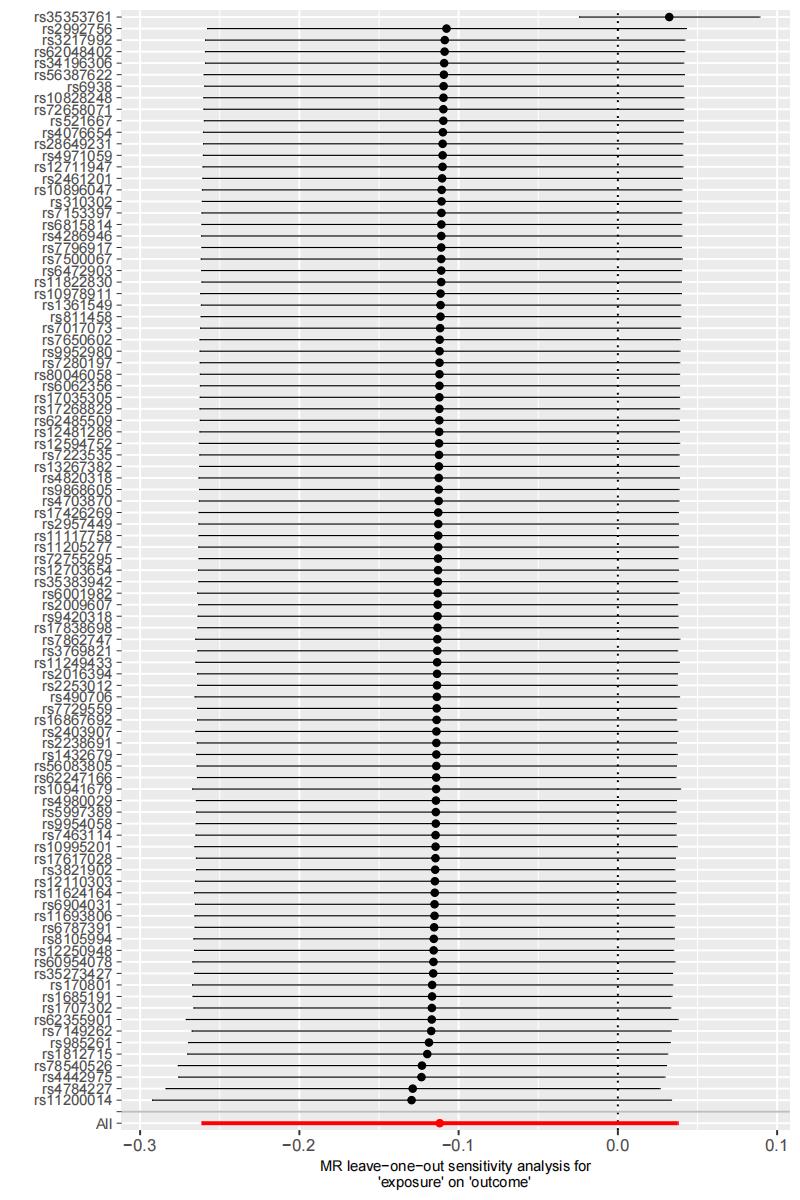


1. ER-negative breast cancer on Ischemic stroke (large artery atherosclerosis)


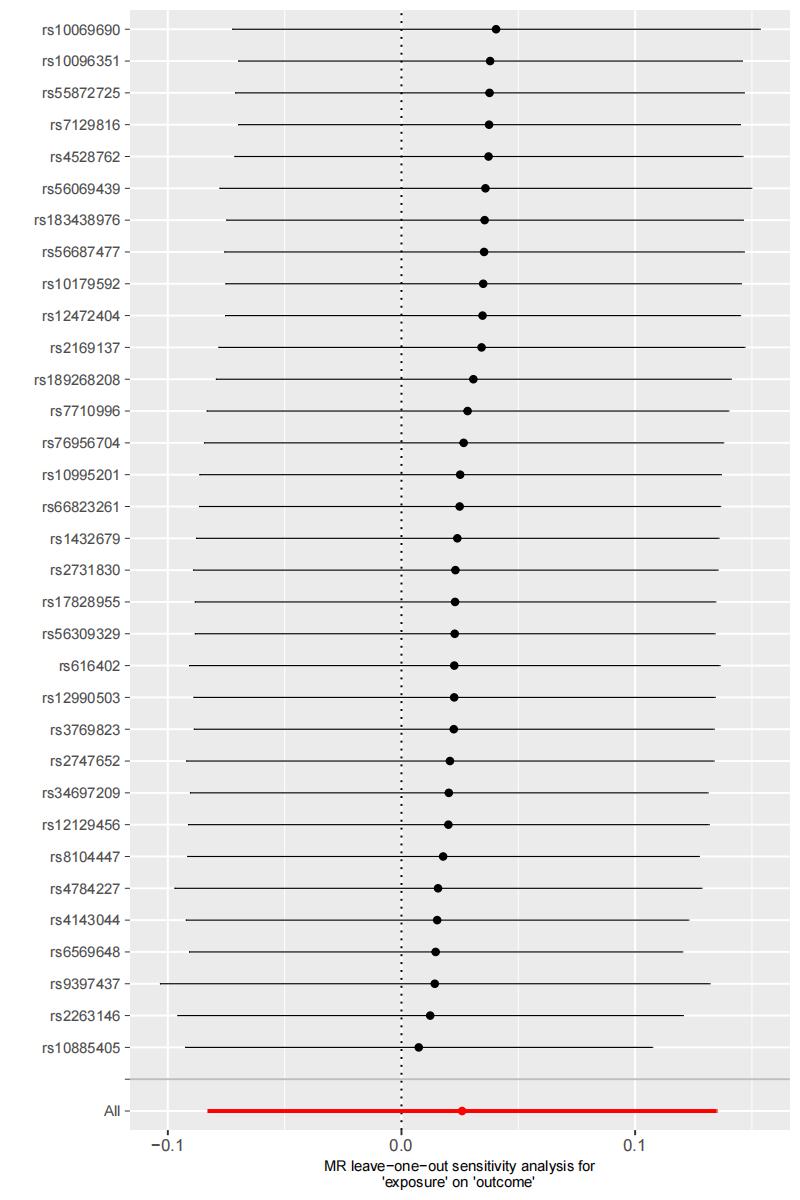


1. Stroke on Breast cancer


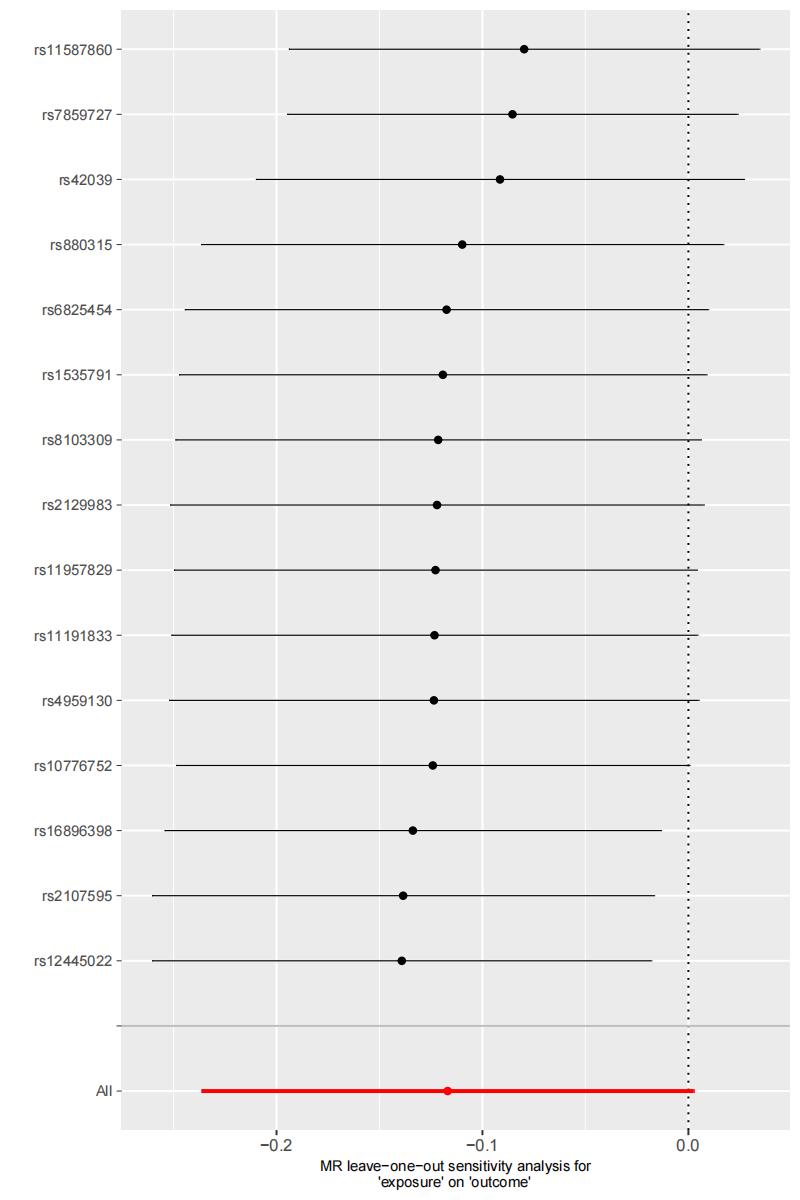


1. Stroke on ER-positive breast cancer


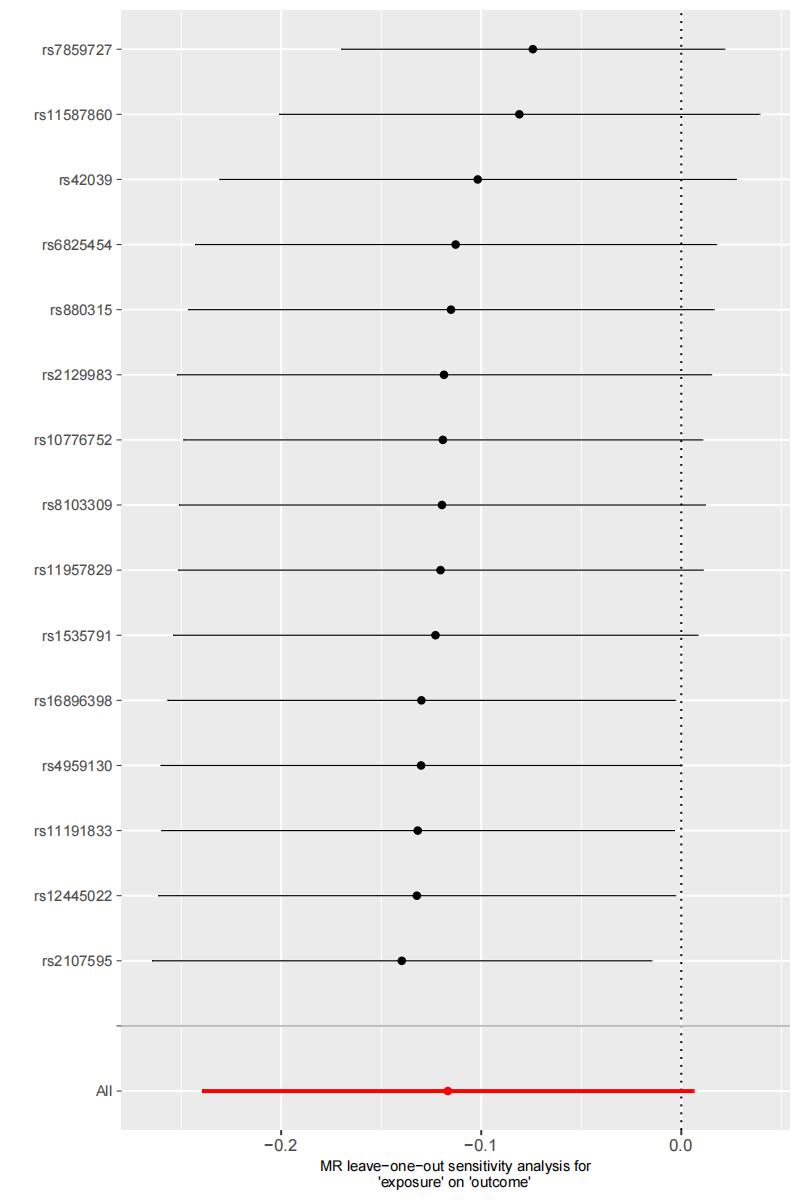


1. Stroke on ER-negative breast cancer


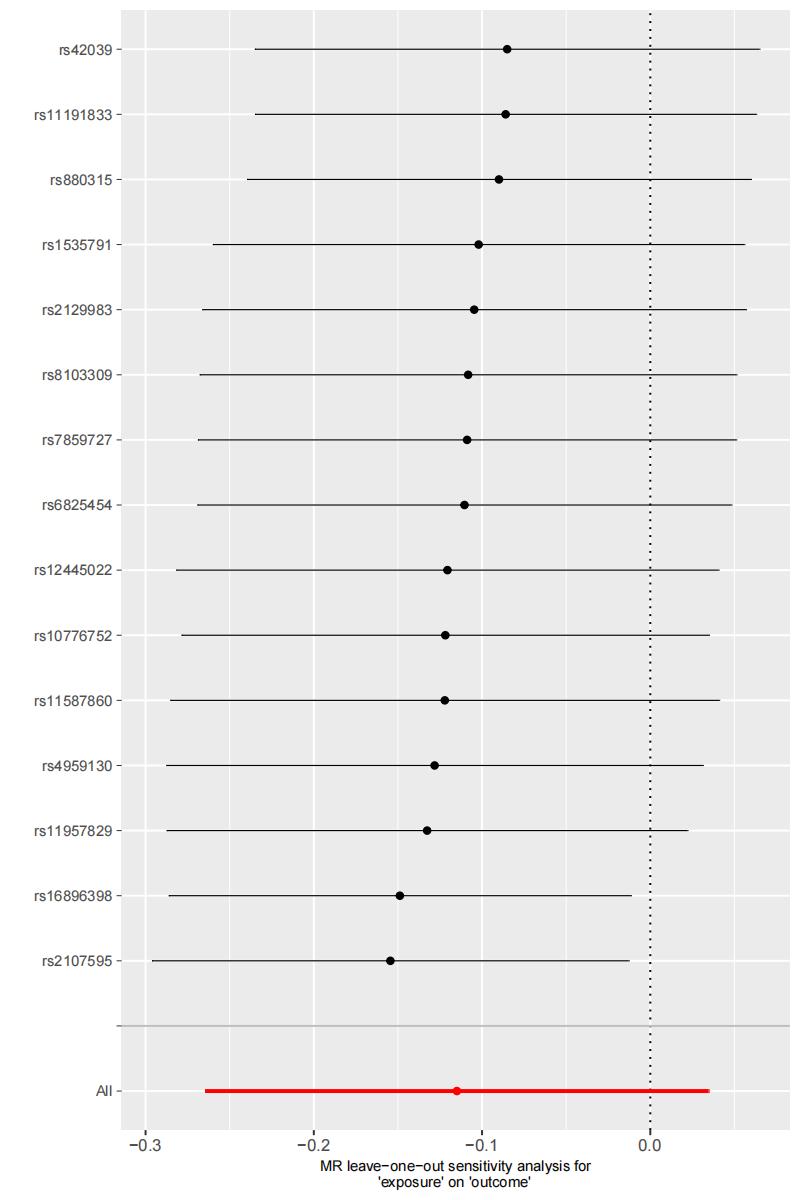


1. Ischemic stroke on Breast cancer


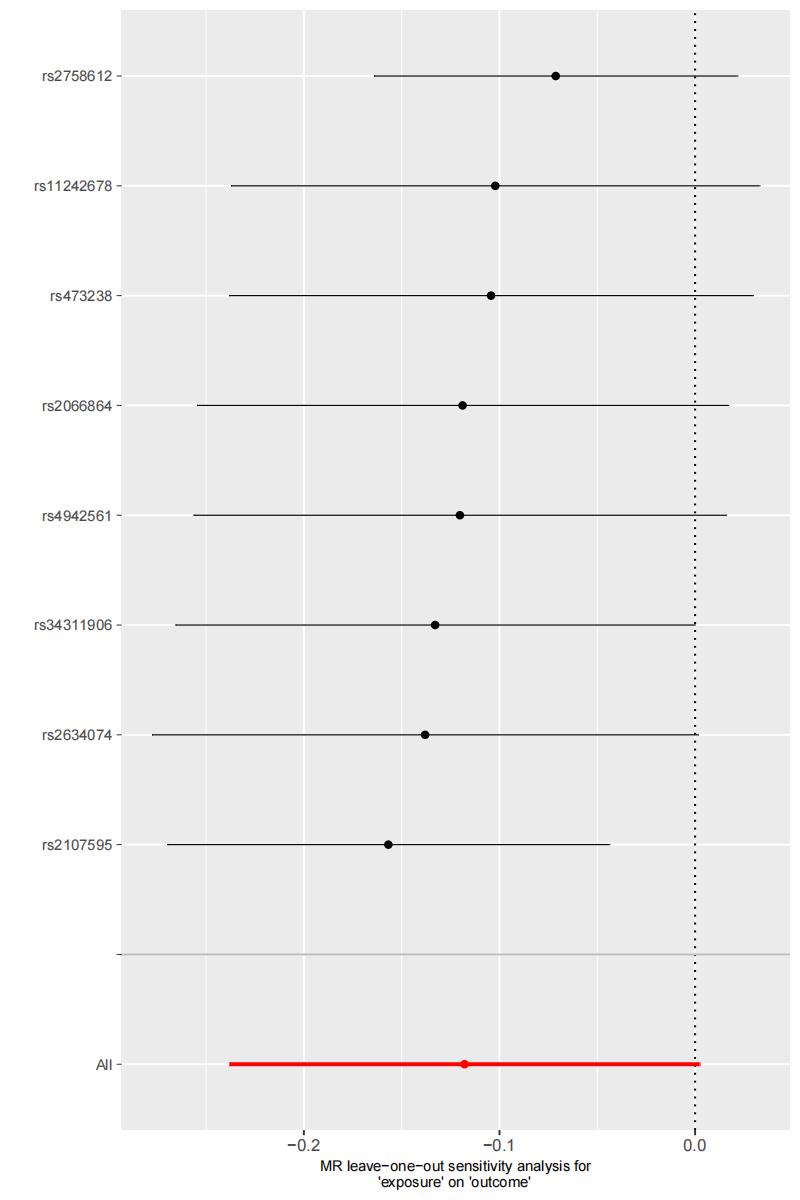


1. Ischemic stroke on ER-positive breast cancer


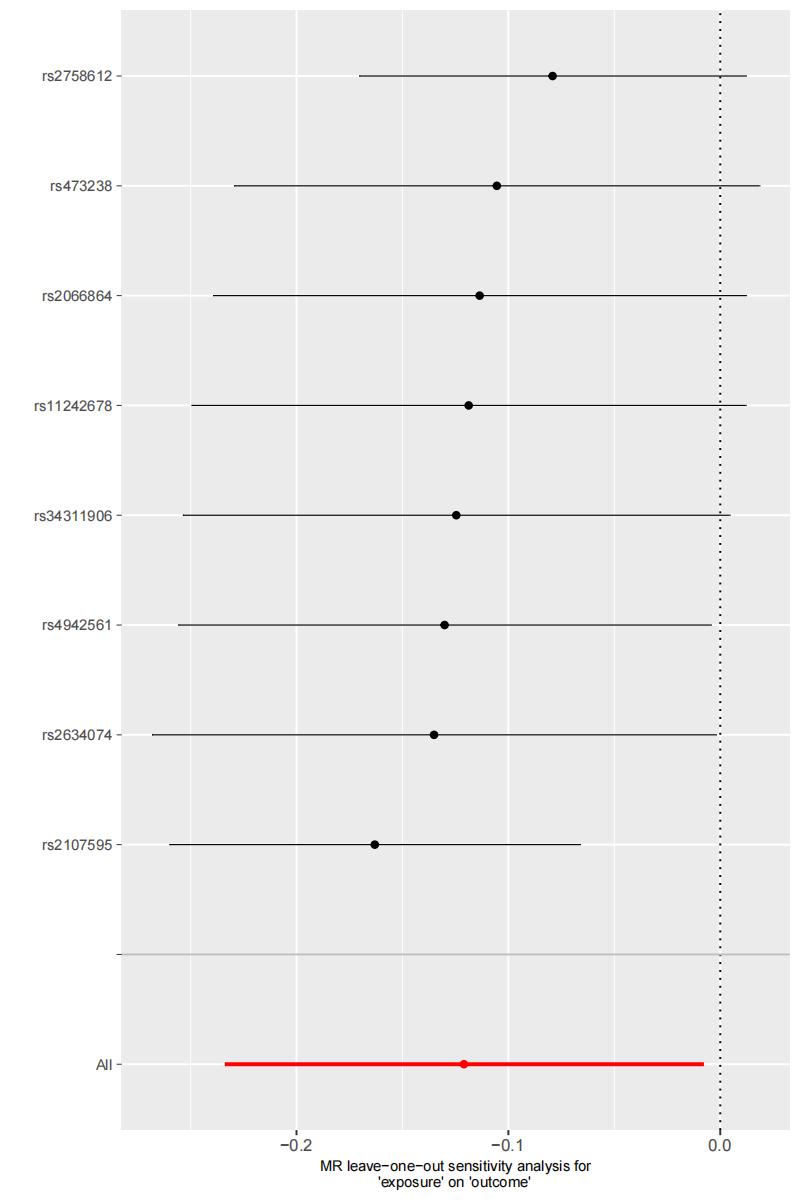


1. Ischemic stroke on ER-negative breast cancer


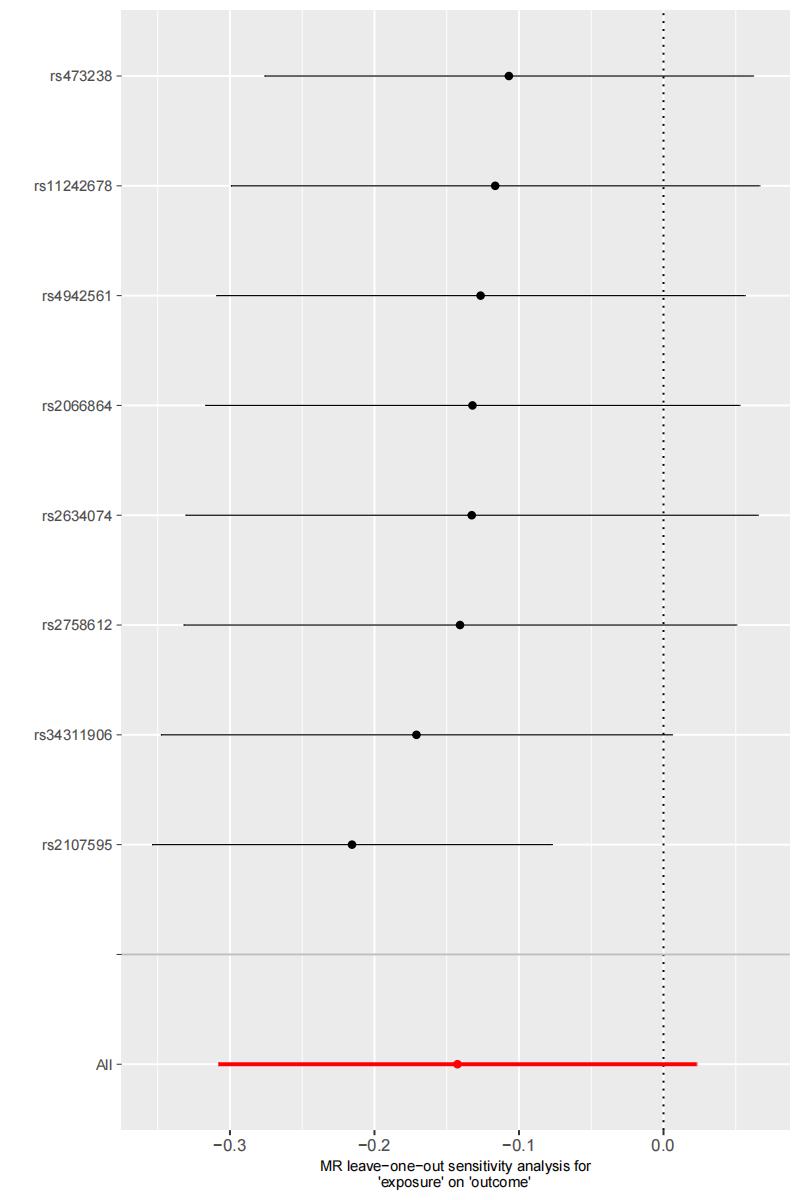


1. Ischemic stroke (cardioembolic) on Breast cancer


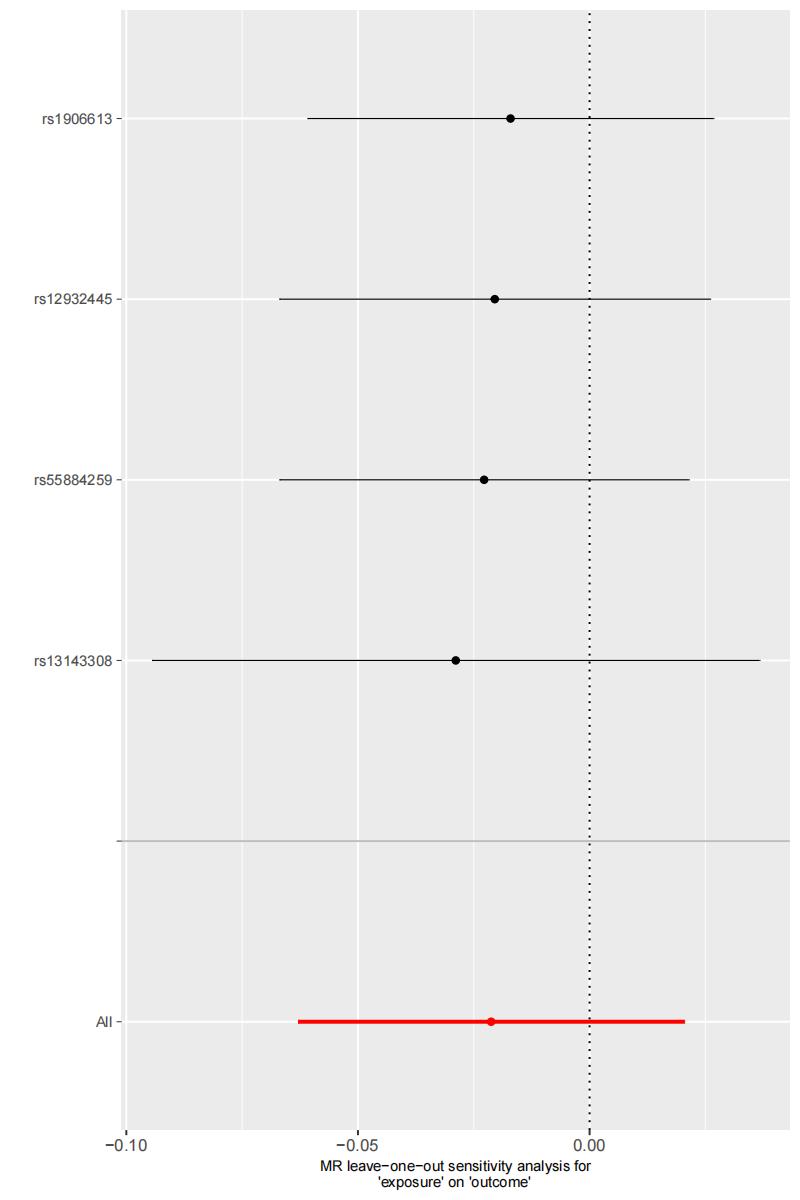


1. Ischemic stroke (cardioembolic) on ER-positive breast cancer


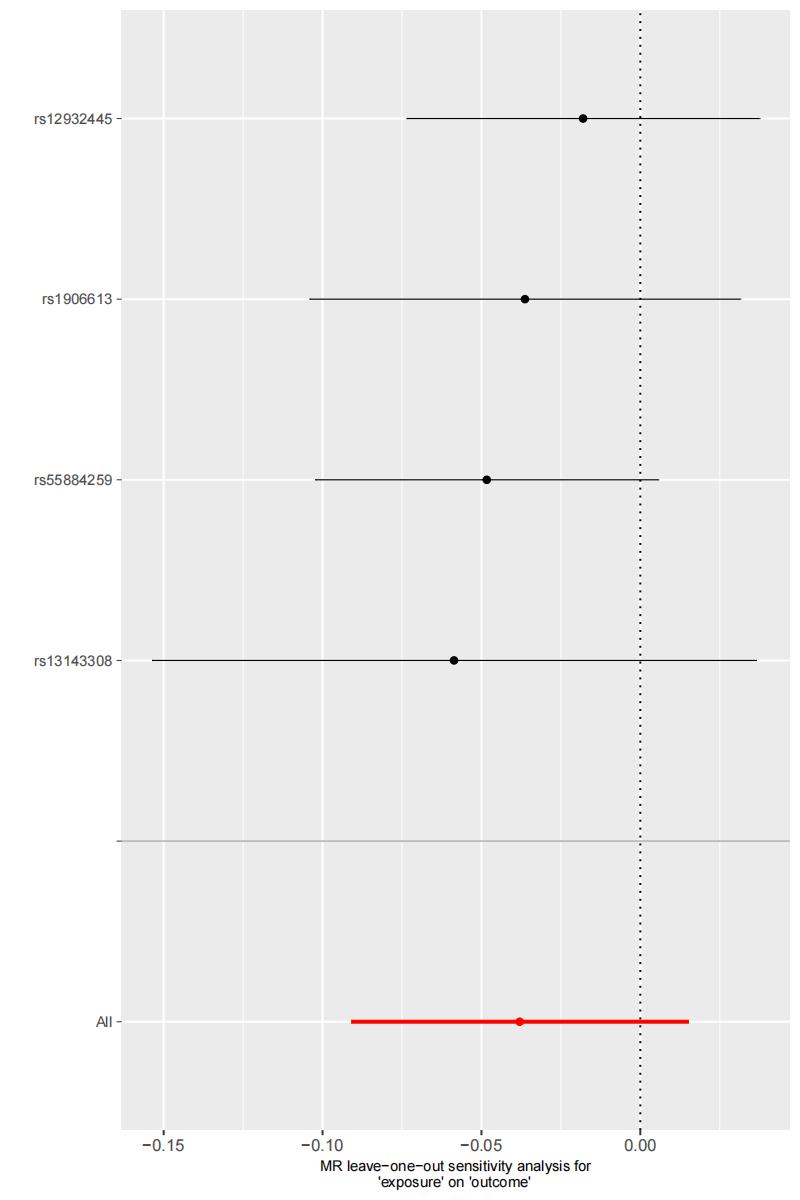


1. Ischemic stroke (cardioembolic) on ER-negative breast cancer


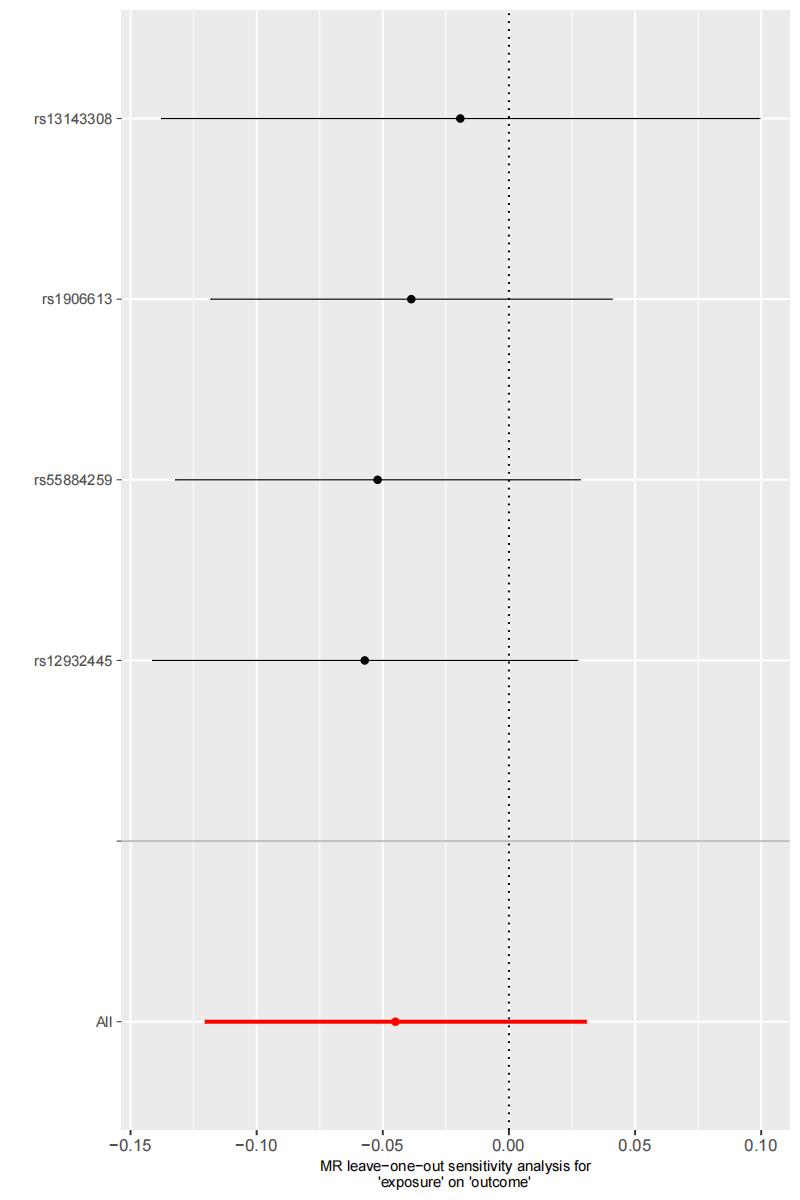


1. Ischemic stroke (large artery atherosclerosis) on Breast cancer


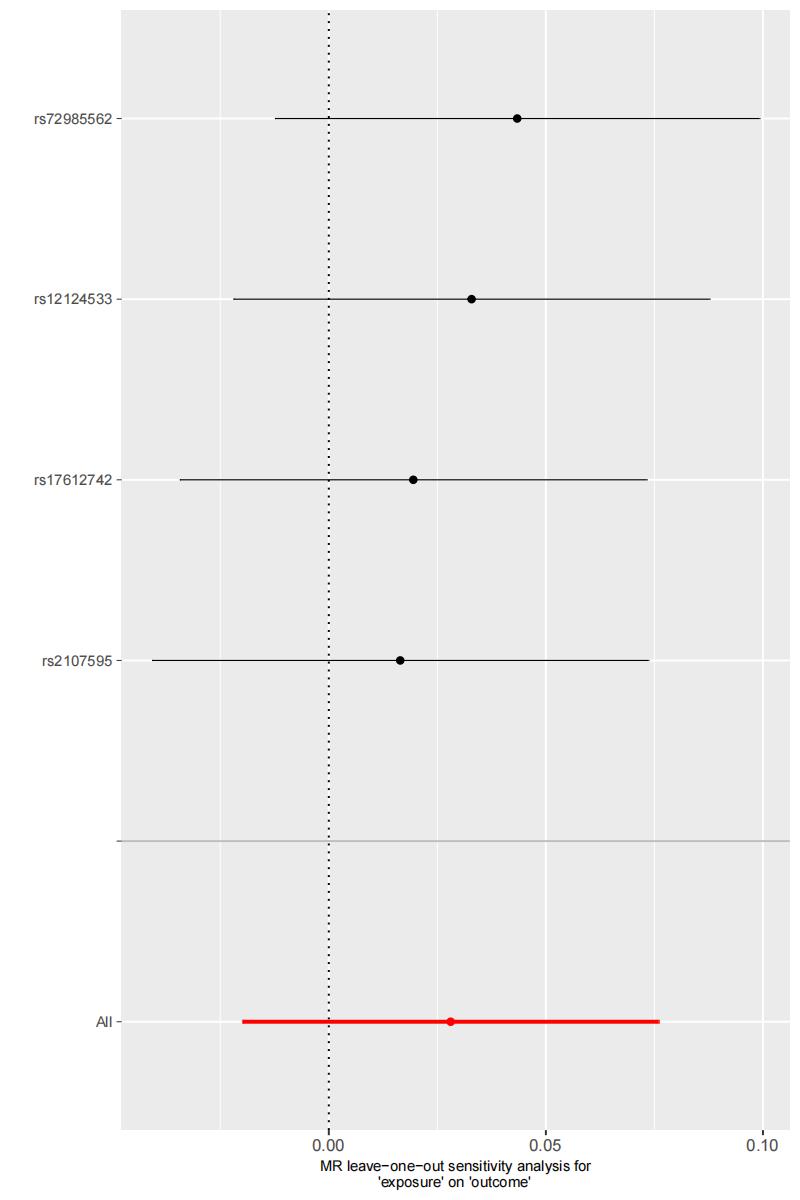


1. Ischemic stroke (large artery atherosclerosis) on ER-positive breast cancer


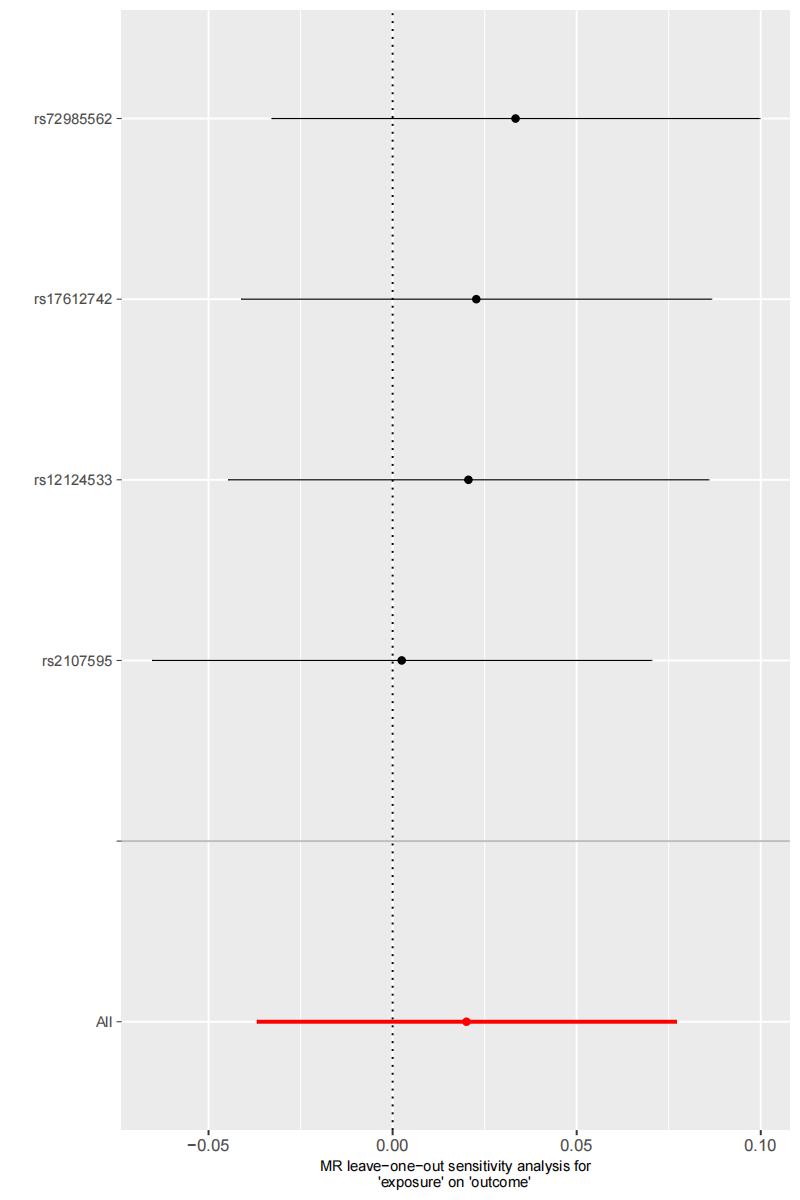


1. Ischemic stroke (large artery atherosclerosis) on ER-negative breast cancer


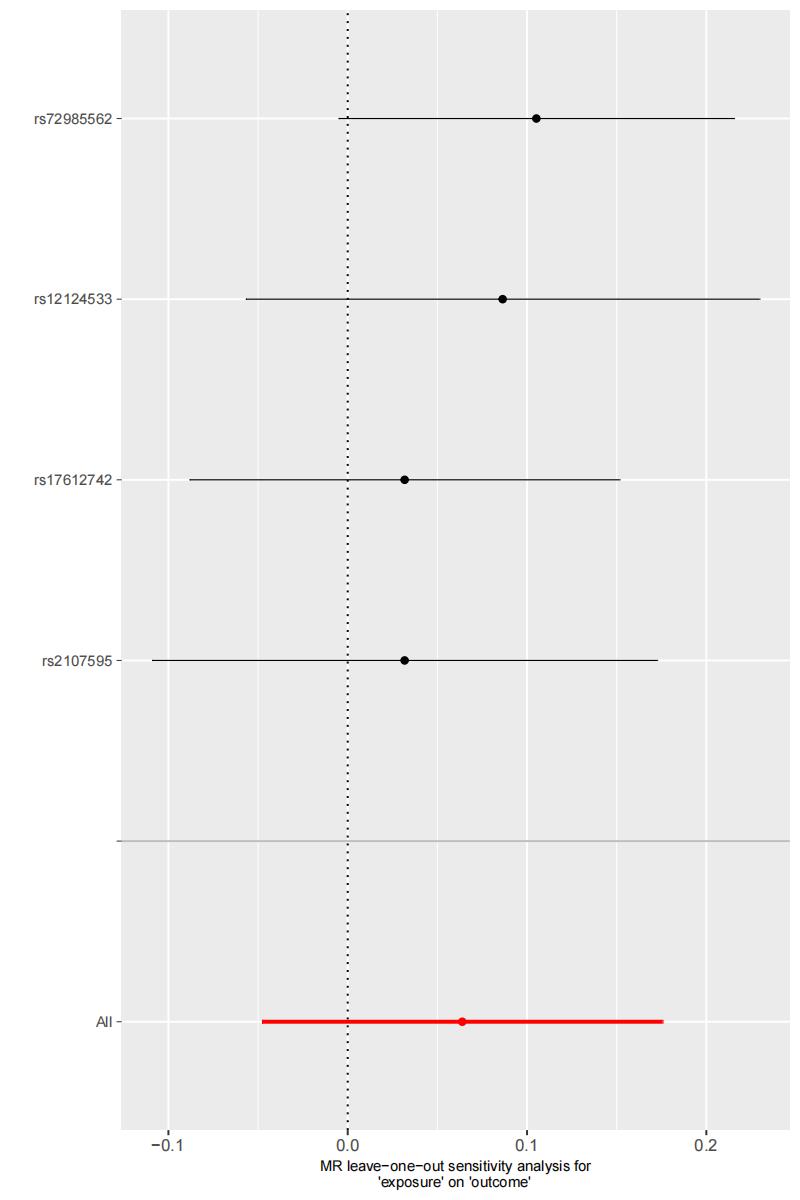

Supplement: Supplementary file 3 [file medi-102-e35037-s003.docx]
